# Characterization of goat prions demonstrates geographical variation of scrapie strains in Europe and reveals the composite nature of prion strains

**DOI:** 10.1038/s41598-019-57005-6

**Published:** 2020-01-08

**Authors:** Romolo Nonno, Alba Marin-Moreno, Juan Carlos Espinosa, Christine Fast, Lucien Van Keulen, John Spiropoulos, Isabelle Lantier, Olivier Andreoletti, Laura Pirisinu, Michele A. Di Bari, Patricia Aguilar-Calvo, Theodoros Sklaviadis, Penelope Papasavva-Stylianou, Pier Luigi Acutis, Cristina Acin, Alex Bossers, Jorge G. Jacobs, Gabriele Vaccari, Claudia D’Agostino, Barbara Chiappini, Frederic Lantier, Martin H. Groschup, Umberto Agrimi, Juan Maria Torres, Jan P. M. Langeveld

**Affiliations:** 1 Istituto Superiore di Sanità, Department of Food Safety, Nutrition and Veterinary Public Health, Rome, Italy; 2 Centro de Investigación en Sanidad Animal, CISA-INIA Madrid, Spain; 3 Institute of Novel and Emerging Infectious Diseases, Friedrich-Loeffler-Institute, Greifswald-Isle of Riems, Germany; 4 Wageningen BioVeterinary Research, Lelystad, the Netherlands; 5 Animal and Plant Health Agency, New Haw, Addlestone, Surrey, United Kingdom; 6 INRA-Centre Val de Loire, Infectiologie et Santé Publique, Nouzilly, France; 7 UMR INRA ENVT 1225- IHAP, École Nationale Vétérinaire de Toulouse, Toulouse, France; 8 Laboratory of Pharmacology, School of Health Sciences, Department of Pharmacy, Aristotle University of Thessaloniki, Thessaloniki, Greece; 9 Veterinary Services, Nicosia, Cyprus; 10 Istituto Zooprofilattico Sperimentale del Piemonte, Liguria e Valle d’Aosta, Torino, Italy; 11 Centro de Encefalopatías y Enfermedades Transmisibles Emergentes, Facultad de Veterinaria, Universidad de Zaragoza, Zaragoza, Spain

**Keywords:** Neurodegeneration, Infectious diseases, Experimental models of disease, Prion diseases

## Abstract

Bovine Spongiform Encephalopathy (BSE) is the only animal prion which has been recognized as a zoonotic agent so far. The identification of BSE in two goats raised the need to reliably identify BSE in small ruminants. However, our understanding of scrapie strain diversity in small ruminants remains ill-defined, thus limiting the accuracy of BSE surveillance and spreading fear that BSE might lurk unrecognized in goats. We investigated prion strain diversity in a large panel of European goats by a novel experimental approach that, instead of assessing the neuropathological profile after serial transmissions in a single animal model, was based on the direct interaction of prion isolates with several recipient rodent models expressing small ruminants or heterologous prion proteins. The findings show that the biological properties of scrapie isolates display different patterns of geographical distribution in Europe and suggest that goat BSE could be reliably discriminated from a wide range of biologically and geographically diverse goat prion isolates. Finally, most field prion isolates showed composite strain features, with discrete strain components or sub-strains being present in different proportions in individual goats or tissues. This has important implications for understanding the nature and evolution of scrapie strains and their transmissibility to other species, including humans.

## Introduction

Transmissible spongiform encephalopathies (TSEs) or prion diseases include fatal neurological diseases of humans and animals which can have different origin and epidemiology, i.e. acquired, sporadic or familial. Prion diseases are caused by a conformational switch of soluble cellular prion protein monomers (PrP^C^) into misfolded, partially protease-resistant and insoluble prion protein aggregates (PrP^Sc^), which then propagate through an autocatalytic conformational conversion.

TSEs have been reported in several livestock species, such as small ruminants, bovines, cervids and, more recently, camels^1^. Thus, humans may be exposed to TSE infected tissues of animal origin through the food chain. Some of these TSEs in animals, such as chronic wasting disease (CWD) in cervids and classical scrapie in sheep and goats, efficiently spread by natural transmission and are thus infectious under field conditions. So far, there is no proof of zoonotic transmission of scrapie or CWD, although their zoonotic potential remains incompletely characterized^2^. BSE is the only known animal TSE agent with proven transmissibility to man. The chance that BSE may circulate in sheep and goats led to the need to reliably identify BSE outbreaks in small ruminants. In particular, discriminatory methods able to differentiate between scrapie and BSE have been introduced, leading to the identification of BSE in two goats^3–5^.

Prions occur as different strains, which are identified by bioassay experiments in rodents. However, the strain properties are thought to depend on the specific conformation of PrP^Sc^ and the molecular or immunohistochemical phenotypes in the original host species (often referred to as “types”) allow some differentiation. As a notable example, BSE in small ruminants can be discriminated from scrapie by Western blot (WB) analysis of proteinase-K treated PrP^Sc^ (PrP^res^)^6–10^ and by immunohistochemistry (IHC)^11,12^. Classical scrapie, Nor98 (also referred to as atypical scrapie), CH1641 scrapie and BSE are the TSE types which can be recognized in sheep based on WB PrP^res^ banding patterns.

Classical scrapie is the prototypic animal TSE and it is known to be caused by more than one TSE strain^13–19^. CH1641 is a peculiar scrapie strain which has been propagated by experimental transmissions in sheep^20^. CH1641 is characterized by N-terminal PrP^res^ features resembling those of BSE^8,21^, but is distinguishable from BSE by discriminatory immunohistochemistry^22^ and by improved molecular typing approaches^23–26^. Intensified surveillance in the EU allowed to identify molecular and pathological properties reminiscent of experimental CH1641 in naturally affected sheep and goats ;^12,24,25,27,28^ these cases have been often referred to as CH1641-like scrapie. Finally, Nor98 was first detected in 1998 in Norway as a new form of scrapie characterized by distinctive clinical and pathological features^29^, and has been later recognized as a sporadic, putatively spontaneous or poorly transmissible, form of scrapie affecting small ruminants worldwide^30^.

The PrP^res^-based discrimination scheme is of practical relevance, as it provided the basis for BSE surveillance in small ruminants, but is far from representing the whole variability of scrapie strains. Contrary to Nor98 which represents a single prion strain^31^, several strains of classical scrapie in sheep have been documented in UK and France^16–19^, and one in Italy^32^. Unfortunately, goat scrapie strains have been less investigated and very little knowledge has been gathered on scrapie strain diversity in goats, thus limiting the accuracy and reliability of BSE surveillance in goats. However, this knowledge is necessary both for the ability to properly identify BSE in small ruminants and for the understanding of the zoonotic potential of scrapie.

The overall aim of this project was to characterize goat TSEs in order to i) allow a clear-cut discrimination of BSE in goat and ii) investigate the diversity of goat TSE strains in the field in the EU. We selected a panel of EU goat TSE isolates and characterised their molecular and biological properties in different EU labs. While the detailed molecular features of PrP^Sc^ in the chosen goat TSE isolates panel will be reported elsewhere in parallel^33^, we here report the results of the bioassay experiments. Importantly, all experiments were done with aliquots of the same brain macerates, so as to reduce any sampling bias and to allow proper comparison of different animal models. Thus, we compared the transmission properties of the isolates after intracerebral inoculation in a panel of rodent models able to provide the most broad and sensitive approach for strain typing.

## Results and Discussion

### Rodent models display differential susceptibility to goat TSEs

Wild type mice (RIII) and bank voles (Bv109M), as well as several lines of transgenic mice expressing PrP from goat (tg501, hereafter referred to as tg-gtARQ), ARQ-sheep (tg-shpIX, referred to as tg-shARQ), VRQ-sheep (tg338, referred to as tg-shVRQ), bovine (tg110, referred to as tg-bov) or mouse (tga20) were inoculated with the panel of isolates displayed in Table 1. Most of the isolates had the classical scrapie PrP^Sc^ type, hereafter referred to as “21 K”, which can be discriminated from experimental BSE in goat by the lower MM (referred to as “19 K”) and the reduced binding with mAb P4^8^. Two of the isolates from the panel were an Italian goat with atypical scrapie (I15)^34^ and an unusual goat case with 19 K PrP^Sc^ originating from a large scrapie outbreak in an English herd^28^, later shown to have strain properties distinct from BSE^35^ and clearcut CH1641-like biochemical features (UKB2)^33^.

**Table 1:** Geographic origin, PrP genotype and PrP^Sc^ type of goat TSE isolates included in the study.

Country of origin	Original ID	Code	PrP genotype*	PrP^Sc^ type^§^
Italy	114921/1/1	I2	240PP	Classical scrapie
Italy	121429/1/1	I3	240PP	Classical scrapie
Italy	128710/1/1	I4	211QR, 240PS	Classical scrapie
Italy	17646/1/1	I5	240PP	Classical scrapie
Italy	87016/1/1	I15	154RH; 240PS	Nor98
Italy	85788/1/1	I7	240PP	Classical scrapie
Italy	85792/1/1	I9	143HR, 240PS	Classical scrapie
Italy	117463/1/1	I11	240PS	Classical scrapie
Italy	144508/1/1	I12	240PS	Classical scrapie
Netherlands	577227	N1^	143HR, 240PS	Classical scrapie
Netherlands	586632-32	N2^	143HR, 240PS	Classical scrapie
Netherlands	586632-33	N3^	240PP	Classical scrapie
France	CP40	F2**	240PS	Classical scrapie
France	1028	F3	240PP	Classical scrapie
France	2119	F6	240PS	Classical scrapie
France	2143	F10	240PS	Classical scrapie
France	2154	F11°	142IM, 240PP	Classical scrapie
France	9041	F14	142IM, 240PS	Classical scrapie
France	9135	F16	240PS	Classical scrapie
Spain	C-163P	S2	240PS	Classical scrapie
Spain	C-645P	S3	240PP	Classical scrapie
Greece	1658	G1	240PP	Classical scrapie
Greece	1663	G2	240PP	Classical scrapie
Greece	1676	G3	143HR, 240PP	Classical scrapie
Greece	6989	G4	240PP	Classical scrapie
Cyprus	Zyp13	C1	240PP	Classical scrapie
Cyprus	Zyp21	C2	240PP	Classical scrapie
Cyprus	Zyp27	C3	240PP	Classical scrapie
UK	BR, G08-1469	UKB2^^	127GS, 240PP	CH1641-like^§§^
UK	BR, G08-1475	UKA2^^	240PS	Classical scrapie
UK	BR, G08-1446	UKD2^^	127GS, 240PP	Classical scrapie
UK	BR, G08-1460	UKC2^^	240SS	Classical scrapie
France	1075	gtBSE^#^	211RQ, 240PS	BSE

*Only amino acid variations are reported, indicated as the codon number followed by the letter codes of the two amino acids at that position. As the P-to-S PrP polymorphism at codon 240 is exceedingly frequent in EU goats, the amino acids at this position are reported for all goats. ^§^ The PrP^Sc^ type was determined by analyzing the same brain macerates here used for transmission studies^33^. ^§§^ This case was found to have unusual 19 K PrP^Sc^ type in the brain^28^, but strain properties distinct from BSE ;^35^ we later identified CH1641-like PrP^Sc^ properties in this isolate^33^. ^ and ^^ respectively isolates deriving from the same farm. ** Experimentally infected with sheep scrapie by the intracerebral route. ° Experimentally infected with goat scrapie by the oral route. ^#^ Experimentally infected with cattle BSE by the intracerebral route.

The details of rodent models and their overall ability to propagate goat prions are presented in Table 2; the individual attack rates and survival times of all inoculated rodent lines for 33 isolates are reported in Supplementary Table S1. Overall, the transmission rate varied across animal models, as determined by the number of isolates that induced disease in more than 20% of recipient animals (Table 2). Tg-gtARQ and tg-shARQ, which were permissive to all isolates, showed the highest ability to propagate different goat prions, followed by tg-shVRQ. Rodent models expressing bank vole, mouse and particularly bovine PrP were more selective, allowing to propagate only a subset of goat isolates (77%, 72% and 60%, respectively). The overall transmission rate was strongly related to the degree of PrP sequence homology between donor and recipient species, being higher in rodent models expressing small ruminant PrPs than in those with heterologous PrPs. In contrast, the PrP^C^ expression level did not give *per se* a major contribution to the transmission rate, as previously shown by titration experiments^36^. This is well exemplified in tga20 mice, which were as permissive to infection as wild type mice although expressing ten-fold levels mouse PrP^C^. The survival time induced by the different isolates was highly variable, so that in all models either short, around 200 days post inoculation (dpi), or long, >500 dpi, survival times were observed (Table 2).

**Table 2:** Main features of rodent models and overall outcome of transmission experiments (data are derived from Supplementary Table S1).

Species	Line	Name in the paper	PrP sequence	PrP expression level	% positive transmissions*	Mean Inc. time (range)
Mouse	tg501	tg-gtARQ	goat/sheep-ARQ	2×	100% (26/26)	221–>650
Mouse	tgshpIX	tg-shARQ	goat/sheep-ARQ	4×	100% (32/32)	169–531
Mouse	tg338	tg-shVRQ	sheep-VRQ	8×	93% (27/29)	173–745
Mouse	tg110	tg-bov	Cattle	8×	60% (15/25)	195–649
Mouse	tga20	tga20	mouse PrP^a^	10×	72% (13/18)	177–447
Mouse	RIII	RIII	mouse PrP^a^	wild type	72% (18/25)	363–971
Bank vole	Bv109M	Bv109M	vole 109M	wild type	77% (20/26)	173–584

*number of inocula giving attack rate >20%/number of inocula tested in that particular model.

For the subsequent analyses a subset of 20 isolates was selected (Table 3), excluding those isolates which were known to harbour low levels of PrP^Sc^ or which were not inoculated in all rodent models. This allowed us to reduce confounding factors due to different infectious titres among the isolates and to compare more easily the transmission properties of the different isolates across rodent models. Additionally, tga20 have been excluded from the subsequent analyses, as these mice were inoculated with only 18 out of the 33 isolates. Of note, the results in tga20 were much overlapping with those in RIII mice (Supplementary Table S1). Among the 20 selected isolates (Table 3), BSE in goat, the CH1641-like UKB2 and 11 classical scrapie isolates gave efficient transmissions (i.e. with attack rate higher than 20%) in all rodent models. The remaining 7 isolates, including the atypical scrapie I15 and 6 classical scrapie isolates, failed to transmit, or did so very inefficiently, in one or more recipient rodent models. Atypical scrapie showed the lowest ability to adapt and propagate on different PrP species, being able to transmit in transgenic mice carrying small ruminant PrPs, but not in tg-bov, RIII and Bv109M. In contrast, BSE in goat transmitted consistently in all recipient models with full attack rate, showing the shortest survival times in tg-bov and tg-shARQ mice. These results confirm the known broad adaptability of the BSE strain^37^, which is supposedly one of the reasons which allowed this bovine prion strain to cross the human species barrier.

**Table 3:** Transmission features of 20 selected goat TSE isolates in the different rodent models.

TSE isolates		tg-gtARQ			tg-shARQ			tg-shVRQ			tg-bov			RIII			Bv109M		
Country of origin	ID	AR	ST±SD	PrP^Sc^ type	AR	ST±SD	PrP^Sc^ type	AR	ST±SD	PrP^Sc^ type	AR	ST±SD	PrP^Sc^ type	AR	ST±SD	PrP^Sc^ type	AR	ST±SD	PrP^Sc^ type
France	gtBSE	6/6	346±16	19K	12/14	230±59	19/21K	6/6	657±47	19K	6/6	260±14	19K	15/15	363±27	19K	8/8	584±88	19/21K
Italy	I2	6/6	526±123	21K	4/11	410±208	21K	6/6	745±26	21K	1/6	453	21K	0/13			11/11	226±48	21K
Italy	I3	4/4	644±14	21K	6/6	471±74	21K	8/8	641±103	21K	1/7	464	21K	0/13			11/11	222±27	21K
Italy	I15	6/6	552±78*	7K	4/4	354±44	7K	5/5	208±39	7K	0/6			0/13			0/10		
Italy	I12	4/4	591±42	21K	10/12	420±99	21K	3/3	595±173	21K	0/6			0/14			10/10	173±23	21K
N-lands	N1	4/4	437±44	21K	12/13	249±13	21K	6/6	540±78	21K	3/3	649±9	19K	10/10	439±15	21K	6/12	423±134	21K
N-lands	N3	6/6	463±21	21K	11/14	270±14	21K	5/5	266±79	21K	2/6	324;703	19K	15/15	460±21	21K	2/9	476; 894	21K
France	F2	5/5	239±21	21K	11/11	177±25	21K	5/5	173±16	NA	4/5	342±163	19K	2/13	900, 900	NA	2/2	216; 624	21K
France	F3	6/6	287±14	21K	13/14	208±26	21K	6/6	224±37	19K	3/6	290±48	19K	15/15	300±36	21K	5/11	526±276	21K
France	F6	4/4	468±15	21K	13/14	235±37	19/21K	6/6	285±124	19K	6/6	523±166	19K	13/13	471±20	21K	6/11	436±252	19/21K
France	F10	6/6	449±19	21K	11/11	251±65	21K	6/6	284±40	21K	4/4	556±68	19K	15/15	430±11	21K	4/9	276±93	21K
France	F11	6/6	>650	21K	7/9	266±36	21K	5/5	351±30	21K	0/6			11/11	417±39	21K	2/11	348; 674	21K
France	F16	3/3	431±22	21K	5/9	238±78	21K	6/6	413±88	21K	3/6	348±58	21K	6/8	971±66	21K	15/15	193±20	21K
Spain	S2	6/6	228±15	21K	14/15	222±44	19/21K	6/6	198±26	19K	6/6	384±149	19/21K	11/11	475±31	21K	6/13	459±181	19/21K
Spain	S3	6/6	221±16	21K	13/15	210±32	21K	6/6	195±4	19K	5/5	271±19	19K	14/14	451±11	21K	5/9	384±210	19/21K
Greece	G3	4/4	466±35	21K	6/6	292±53	21K	5/5	540±57	19K	0/6			13/13	477±21	21K	0/4		
Cyprus	C1	4/4	483±15	21K	13/13	295±15	21K	5/5	453±65	19K	2/2	>650	19K	15/15	437±13	21K	9/14	292±86	21K
Cyprus	C2	5/5	475±31	21K	13/15	279±54	21K	6/6	459±70	19K	2/5	>650	19K	14/14	435±8	21K	8/11	277±47	21K
UK	UKB2	7/7	345±19	19/21K	6/7	169±34	19/21K	6/6	174±6	19K	7/7	205±12	19K	13/13	429±33	21K	9/11	481±108	19/21K
UK	UKA2	5/5	245±36	19/21K	10/10	231±59	21K	5/5	300±168	19K	5/5	255±69	19K	15/15	418±22	21K	17/18	255±86	19/21K

*Previously reported in ;^40^ in that paper the 154 H/Q genotype of the atypical/Nor98 case I15 was erroneously assigned as wild type.

No obvious correlation was identified between the presence of additional PrP polymorphisms and poor transmissibility in one or more rodent models (Supplementary Table S1). Furthermore, no relation appeared between lack of transmissibility in some rodent models and PrP^Sc^ quantities in the goat brain macerate as estimated by triplex-WB^33^. Overall, these findings show that the transmission rate in different rodent models did not depend simply on the overall low susceptibility of a given model, the low infectious titer or PrP^res^ level of given isolates or the PrP genotype of the donor goat, but reflected different biological properties of the TSE isolates instead. The linkage between the biological properties of prion isolates and their ability to propagate on different PrP sequences can be explained within the conformational selection model of prion strains^38^. Thus, rodent models expressing PrP species homologous to the donor species were able to propagate the different and dominant PrP^Sc^ conformers which characterize isolates with different biological properties, while those expressing heterologous PrP species selectively propagated only those conformations, if any, which can be adopted by the recipient PrP^C^.

### Categorization of goat TSE isolates shows geographical patterns

The above data clearly suggest that the transmission rate in a panel of different rodent models reflects the biological properties of goat TSE isolates. Furthermore, we found that the survival times varied too, with isolates showing either long or short survival time depending on their specific interaction with the recipient rodents (Table 3). Since both the survival time and attack rate yield information on the transmissibility, we sought to incorporate them in a single parameter as a measure of the transmission efficiency (TE) (see methods).

**Figure 1:**
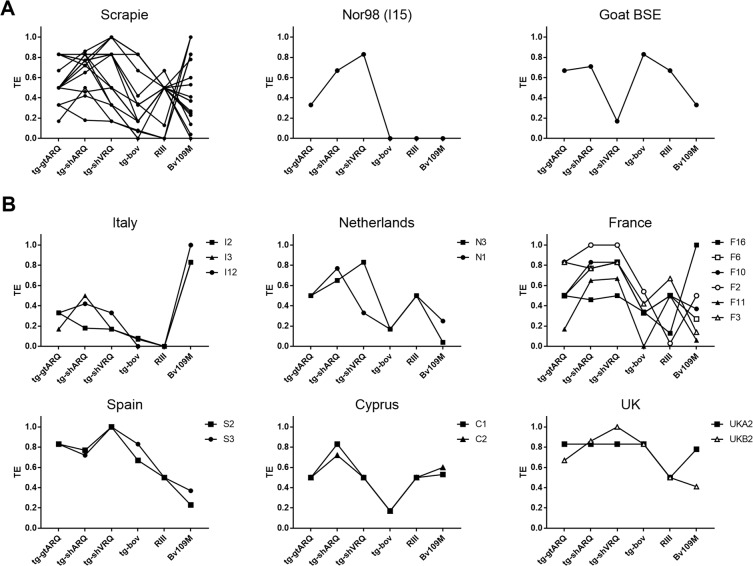
Transmission efficiency (TE) of goat TSE isolates across all rodent models. Graphs depicting TE profiles of the goat isolates reported in Table 3. TE profiles were built by connecting TE values obtained with the same goat isolate in different rodent models. (**A**) TE profiles grouped according to the TSE type, as indicated on the top of each graph. (**B**) TE profiles of classical scrapie isolates according to their country of origin. Individual TSE isolates are referred to as in Table 1.

**Figure 2:**
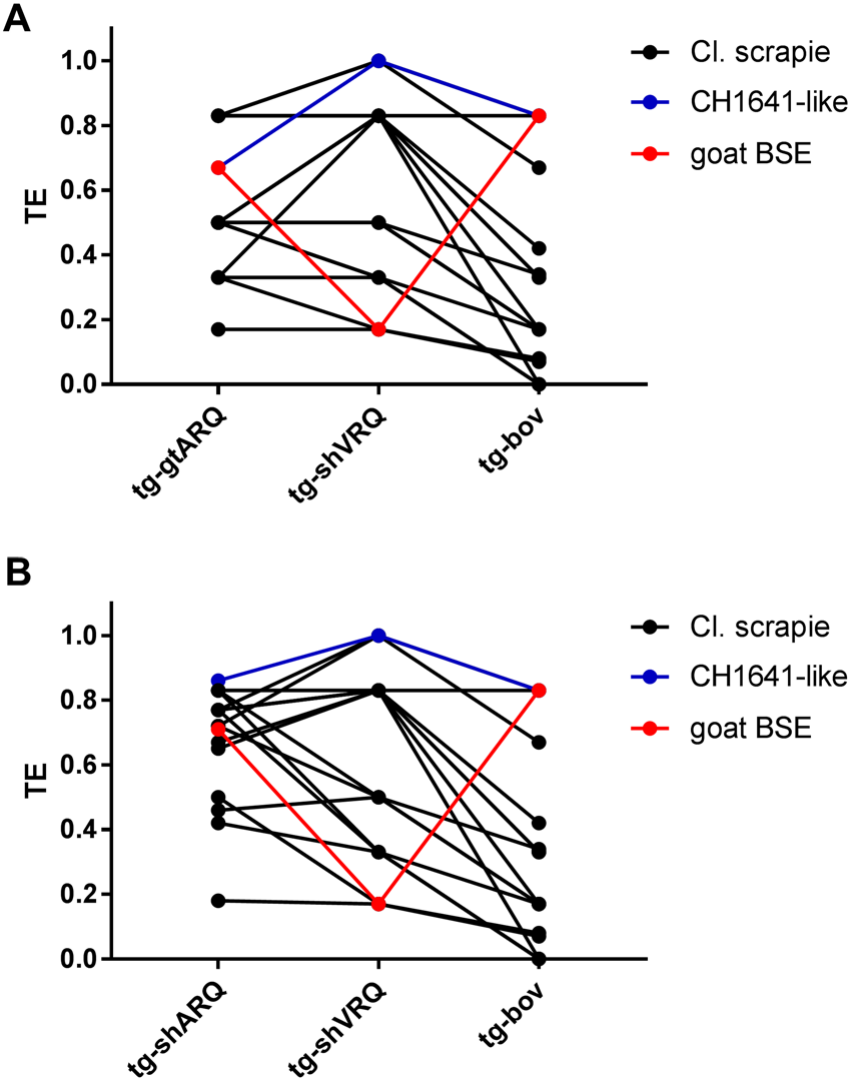
Discrimination of goat BSE from natural goat TSE isolates based on the transmission efficiency in three rodent models. (**A**) Graph showing the TE profiles of goat TSE isolates in tg-gtARQ, tg-shVRQ and tg-bov. The TSE isolates are represented with different colours according to their PrP^Sc^ type (legend on the right). Note that goat BSE could be discriminated from all scrapie isolates, including the CH1641-like, because of the lower transmission efficiency in tg-shVRQ mice compared with both, tg-gtARQ and tg-bov mice. (**B**) The same features are shown in B, where tg-shARQ mice are included instead of tg-gtARQ mice.

By drawing graphs with the TEs of the isolates in different models, it was possible to represent the overall outcome of a given isolate as a transmission profile, which allowed a comparison of the different isolates across all models (Fig. 1). It showed that goat BSE and Nor98 had distinctive transmission profiles, different from each other and from all scrapie isolates (Fig. 1A). Goat BSE was characterized by being the only TSE isolate with lower TE in tg-shVRQ and Bv109M than in any other rodent model. Most importantly, we found that a combination of 3 rodent models, tg-gtARQ or tg-shARQ, tg-shVRQ and tg-bov mice, allowed a clear-cut discrimination of experimental goat BSE from all field scrapie isolates (Fig. 2).

Classical scrapie isolates showed a strong variability of transmission profiles, suggesting the presence of different scrapie strains. Grouping scrapie isolates by country of origin (Fig. 1B) showed that their variability correlates, at least in part, with their geographical origin. In several instances, isolates from the same country had transmission profiles very similar among them and distinct from other countries (see for example graphs for Italy, Cyprus and Spain in Fig. 1B). Notably, the 3 isolates from Italy and the 2 from Spain were sampled in geographically far distant regions, indicating that specific biological properties could be relatively uniform in some countries. In contrast, different transmission profiles were evident among scrapie isolates from France (Fig. 1B). Strikingly, 2 isolates from the same goat herd in the Netherlands and sharing the same PrP^Sc^ type had different behaviour when comparing their TE in tg-shVRQ mice (Fig. 1B).

**Figure 3:**
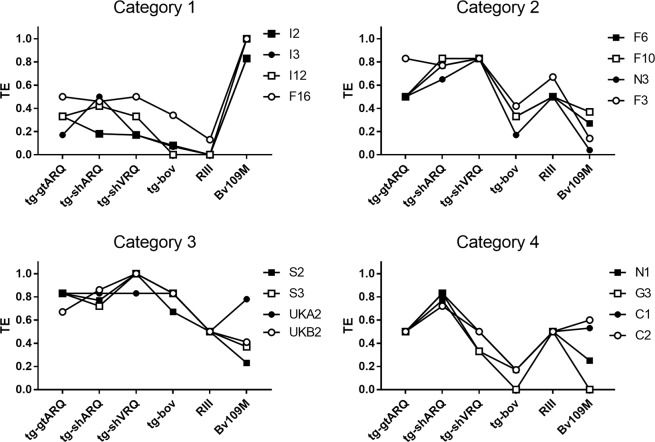
Categories of natural scrapie isolates according to their TE profiles. All natural TSE isolates except the atypical scrapie I15 were grouped according to the similarity of their TE profiles, resulting into 4 categories. Each graph depicts the TE profiles of the isolates belonging to the same category, as reported on the top of each graph. Individual TSE isolates are referred to as in Table 1.

Building on the strong similarities of the transmission profiles of some isolates, we then grouped all natural scrapie isolates by matching their transmission profiles. This resulted into 4 distinct categories of isolates (Fig. 3). TE variation among categories of isolates and rodent models was analysed by ordinary two-way ANOVA, showing significant variation of TEs depending on (i) the rodent model (18.60% of total variation; p < 0.001), (ii) the category (22.97% of total variation; p < 0.001) and (iii) their interaction (44.75% of total variation; p < 0.001). Category 1 included 3 isolates from Italy and 1 from France which were mainly characterized by a higher transmission efficiency in Bv109M than in any other rodent model. Category 2 comprised 3 isolates from France and 1 from the Netherlands, characterized by having the highest TEs in mice which express small ruminant PrP. Category 3, with 2 isolates from Spain and 2 from UK, was similar to category 2 but had higher TEs in tg-bov; interestingly, this category also included the CH1641-like UKB2 isolate. Finally, category 4 included isolates from Greece, Cyprus and 1 from the Netherlands. This latter category was similar to category 2 but had lower TEs in tg-shVRQ. Category 1 was the most distinct, while more overlap was evident among the other three categories, particularly for TEs in tg-gtARQ, tg-shARQ and RIII mice. Though the transmission profiles of isolates belonging to the same categories were remarkably similar, residual TE variation was evident in some rodent model, such as in Bv109M for categories 3 and 4. This might depend on the relatively coarse analysis that we used to derive the TE values, or might imply further strain variation within some categories.

Overall, these findings show that goat BSE has distinctive biological properties reflecting in a TE profile which can be discriminated from all other goat TSE isolates, including the UKB2 isolate characterised by 19 K PrP^Sc^. On the other hand, the biological properties of natural scrapie isolates appear much variable and consistent with geographical patterns of scrapie strain variation in Europe.

### Molecular characterization of PrP^Sc^ in rodents reveals the composite nature of scrapie isolates

**Figure 4:**
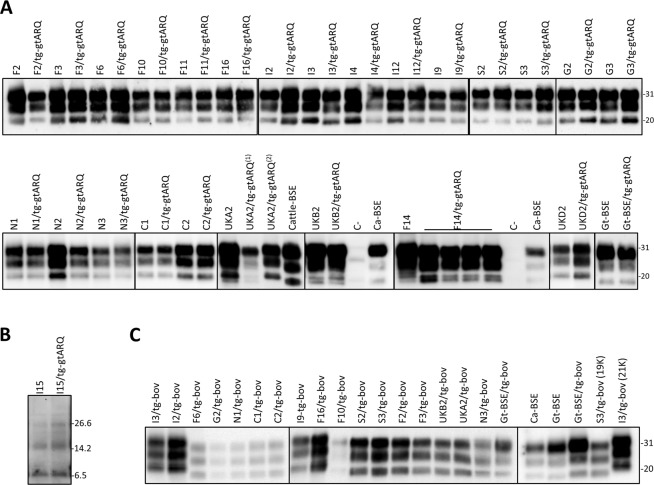
Immunoblots of brain PrP^res^ in goat TSE isolates and in recipient tg-gtARQ and tg-bov mice. (**A**) PrP^res^ of goat classical scrapie isolates, before and after transmission in tg-gtARQ mice. BSE from either cattle (Ca-BSE) or goat (Goat-BSE) have been included for comparison purposes. Healthy goat brain has been included as negative control (**C**). The CH1641-like UKB2 isolate showed a double 19–21 K PrP^res^ unglycosylated band by this WB method, and preserved this signature upon transmission in tg-gtARQ. UKA2 and F14 gave rise to 19–21 K double unglycosylated PrP^res^ similarly to UKB2, although some mice infected with UKA2 exhibited a single 21 K unglycosylated band. The two different PrP^res^ types observed in tg-gtARQ mice after inoculation with the UKA2 isolate are referred as (1) and (2). In all other cases, PrP^res^ in tg-gtARQ mice closely matched that in the corresponding goat isolate. No overlap among PrP^Sc^ types induced by classical scrapie isolates or goat-BSE was thus observed in tg-gtARQ mice, as only goat-BSE resulted in pure 19 K PrP^res^, while classical scrapie isolates propagated as 21 K or mixed 19–21 K PrP^res^. (**B**) PrP^res^ of Nor98/atypical scrapie isolate I15, before and after transmission in tg-gtARQ mice. (**C**) PrP^res^ of goat classical scrapie isolates after transmission in tg-bov mice. Note that several classical scrapie isolates propagated a 19 K PrP^res^ in tg-bov. Goat-BSE led to the propagation of a faithful 19 K PrP^res^, which could be differentiated from the scrapie-derived 19 K by the mostly diglycosylated glycotype. Ca-BSE and Goat-BSE have been included for comparison purposes. In all panels, PrP^res^ was detected with mAb Sha31. The position of molecular mass markers (kDa) is shown on the right of the blots. In all panels the blots are cropped and contrast-enhanced equally across the entire images in order to allow a clear visualization of unglycosylated PrP^res^ in all samples. In panels (**A,C**) the blots are cropped from different blots, as indicated by dividing black lines. The original full-length blots are presented in Supplementary Figs S7 and S8.

The biochemical features of the PrP^Sc^ conformers propagated in the brain of recipient rodents were investigated by WB analysis. Brain tissues from mice and voles were analysed in different laboratories, each using its own WB method. The details of WB results in the different rodent models are described in Supplementary Results. Overall, rodent models propagated three main PrP^Sc^ types, herein referred to as 21 K, 19 K and 8 K based on the apparent MM of their unglycosylated fragment (see Methods paragraph “Definitions and nomenclature”). The most frequent PrP^Sc^ type was the scrapie-like pattern 21 K, which was similar to PrP^Sc^ detected in goats with classical scrapie (Fig. 4), while 19 K had a BSE-like MM (Fig. 4) and was similarly discriminated by decreased binding with N-terminal antibodies such as 12B2 and P4 (Supplementary Figs S1–S3). The third PrP^Sc^ type, 8 K, was detected only in rodent models able to propagate the Nor98 isolate I15, i.e. in tg-gtARQ, tg-shARQ and tg-shVRQ mice. They faithfully reproduced the Nor98 molecular signature (Fig. 4), as previously shown^31,39^. The 8 K PrP^Sc^ type in tg-gtARQ inoculated with the Nor98 I15 isolate had been already reported^40^.

No obvious variation of PrP^Sc^ features, such as the degree of glycosylation or the presence of additional PrP^res^ fragments, has been observed among individual rodents showing 21 K PrP^Sc^. A thorough examination of PrP^Sc^ in RIII mice by triplex WB^41^ (see Supplementary Results) didn’t reveal any variation in the 21 K molecular profile observed after inoculation of isolates with different geographical origin. Furthermore, it showed that all scrapie isolates, including the 19 K UKB2, resulted in 21 K PrP^Sc^ in RIII mice (Supplementary Fig. S2).

In contrast to RIII mice, the propagation of 19 K PrP^Sc^ in all other rodent models was not restricted to experiments with goat BSE, as a similar 19 K PrP^Sc^ type was also derived from the 19 K UKB2 isolate and from a subset of classical scrapie isolates. In most of these cases the scrapie-derived BSE-like PrP^Sc^ type could still be discriminated from BSE PrP^Sc^. The 19 K PrP^Sc^ type propagated upon inoculation of scrapie isolates in tga20 (not shown) and tg-bov mice (Fig. 4) was much less glycosylated than 19 K derived from goat BSE, in agreement with findings in the same transgenic mouse line inoculated with sheep scrapie and BSE^42^. In tg-gtARQ mice, some scrapie isolates induced PrP^Sc^ with double 19/21 kDa unglycosylated bands, instead of pure 19 K as observed in goat BSE (Fig. 4). In Bv109M, 19 K derived from scrapie cases was frequently, but not invariably, accompanied by an additional 13 kDa C-terminal PrP^res^ fragment (Supplementary Fig. S4), reminiscent of that observed in sheep with CH1641^25^. Despite the above-mentioned differences, in Supplementary Table S1, Tables 3 and 4 all PrP^Sc^ types with a MM of the unglycosylated band BSE-like are indicated as 19 K, whereas 19/21 kDa double PrP^Sc^ bands and experiments replicating alternatively 21 K or 19 K in individual recipients are indicated as 19 K/21 K.

**Table 4:** Summary of the PrP^Sc^ types reproduced by rodent models (data are derived from Supplementary Table S1).

Rodent model	PrP^Sc^ types induced by field TSE cases	% 19 K*	PrP^Sc^ types induced by goat BSE
tg-gtARQ	21 K/19 K/8 K	13%	19 K
tg-shARQ	21 K/19 K/8 K	13%	19 K/21 K
tg-shVRQ	21 K/19 K/8 K	44%	19 K
tg-bov	21 K/19 K	76%	19 K
tga20	21 K/19 K	8%	19 K
RIII	21 K	0%	19 K
Bv109M	21 K/19 K	30%	19 K/21 K

*percentage of transmitted classical scrapie isolates inducing 19 K PrP^Sc^.

The PrP^Sc^ types observed in the different rodent models are summarized in Table 4. Goat BSE induced faithful 19 K PrP^Sc^ type in all recipient rodents; however, a subset of recipient tg-shARQ and Bv109M also propagated 21 K (2/12 and 4/8, respectively). Concerning Bv109M, this finding is in line with previous data showing that 19 K or 21 K could be selectively replicated in individual voles inoculated with two other BSE-related prions, sheep BSE and variant CJD^43^. Similar findings have been observed in transgenic mice overexpressing human PrP^44,45^, which might suggest that PrP overexpression and/or the mouse genetic background could have putatively driven the emergence of 21 K upon inoculation of BSE in tg-shARQ mice. There was general consensus among the different WB methods and rodent models that PrP^Sc^ with BSE-like properties can be propagated after inoculation with some goat scrapie isolates. However, the probability for this to occur strongly depended on the rodent model considered, with as much as 76% and 44% of the scrapie isolates propagating 19 K in tg-bov and tg-shVRQ, respectively. In contrast, 13% and 0-to-8% of the scrapie isolates did so in recipient mice with ARQ or mouse PrP, respectively (Table 4). Furthermore, rodents less able to propagate 19 K from classical scrapie isolates, such as tg-gtARQ and Bv109M, mainly showed mixed 19 K/21 K features. These data strongly suggest that the ability to propagate 19 K from classical scrapie isolates might depend on the PrP sequence of the recipient rodent model. However, other factors such as the PrP expression level^46^ or background genetic factors^43^ might have played a role, too.

In accordance with our findings in tg-bov, the emergence of the 19 K PrP^Sc^ signature has been observed upon transmission of classical scrapie into cattle^47–49^ and in bovine PrP tg mice^35,37,42^. Transmission studies in bank voles suggested that the emergence of 19 K in cattle experimentally infected with scrapie could have been linked to the emergence of CH1641 biological properties^48^. Thus, bovine PrP might selectively propagate the 19 K component even when present in minimal amounts in the starting inoculum. It is more difficult to explain why 19 K was also propagated in transgenic mouse lines expressing small ruminant PrP sequences, also considering that scrapie cases with 19 K PrP^Sc^ are rather uncommon in small ruminants. In the case of tg-shVRQ mice, a recent seminal paper showed that the overexpression of PrP determines the selection of the 19 K strain component in these mice^46^. Furthermore, the 19 K scrapie-derived strain showed strain features indistinguishable from CH1641 in tg-shVRQ^46,50^. Finally, experimental transmission *per se* could have favoured the propagation of the 19 K component, as it has been shown that experimental challenge of sheep with classical scrapie can lead to the emergence of a 19 K CH1641-like component, which was hidden in the homotypic inoculum^51^.

**Figure 5:**
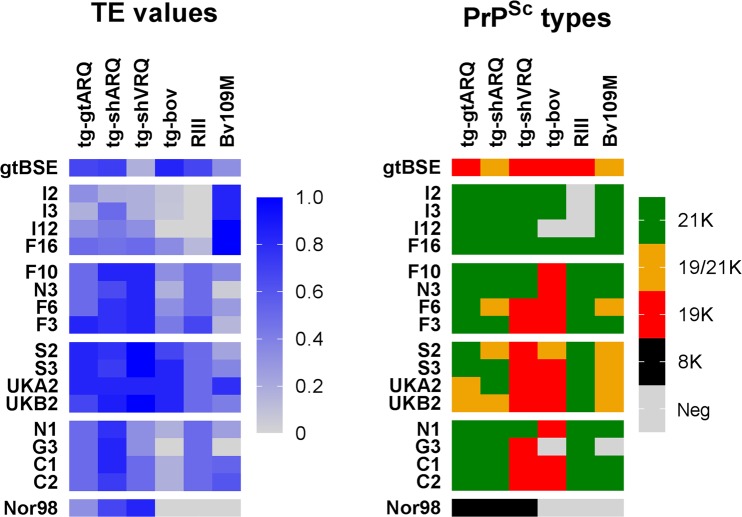
Comparison between PrP^Sc^ types and transmission efficiency of goat TSE isolates in different rodent models. Parallel matrices showing the efficiency of transmission (left graph depicting TE values) and the corresponding PrP^Sc^ types (right graph) propagated by the goat TSE isolates in different rodent models, as indicated on the top of the graphs. In the left panel, TE values are represented by a colour gradient (legend on the right of the graph). In the right panel, PrP^Sc^ types are represented by different colours, according to the legend on the right of the graph. Classical scrapie isolates are ordered according to the TE categories, showing that there is some association between TE-based categories and the emergence of 19 K in one or more recipient models.

We then co-analysed the PrP^Sc^ type and the transmission efficiency in recipient rodent models (Fig. 5). Among all isolates, Goat-BSE only was able to induce 19 K PrP^Sc^ in all recipient rodent models, while the Nor98 isolate I15 invariably induced the 8 K signature. Among scrapie isolates, only those in category 1 were associated with the propagation solely of the 21 K in all rodent models, while all the others induced also the propagation of 19 K in one or more rodent models. Isolates from categories 2-to-4 invariably propagated 19 K in tg-bov, while their ability to induce 19 K as a minor component in tg-shARQ, tg-gtARQ or Bv109M varied depending on the category, being highest in category 3 and absent in category 4. Thus, the ability to induce 19 K might well be correlated with the biological properties of the isolates.

Furthermore, the emergence of 19 K strongly correlated with TE in tg-bov, i.e. isolates with the highest TE in tg-bov were also those inducing 19 K in rodent models less prone to replicate 19 K, such as tg-shARQ, tg-gtARQ or Bv109M. This suggests that the 19 K component isolated in the different models might all represent the same 19 K strain, supposedly CH1641, which is present as a hidden component in different proportions in isolates from different categories, being highest in category 3, lowest in category 4 and absent in category 1. This is in agreement with the finding that the UKB2 isolate, which is CH1641-like in origin^33^, was the most able to induce 19 K in recipient rodents, while other isolates in which the CH1641-like component was hidden, i.e. not detected by WB in the isolate, also induced less 19 K in the recipient models.

Although the transmission efficiency allowed to group isolates by their biological properties, the extent to which these categories equate to specific scrapie strains is difficult to assess. Indeed, the operational definition of prion strains relies on the pathological characterization of serially passaged, rodent-adapted isolates. However, natural scrapie isolates can be frequently mixtures of different strain components or sub-strains instead of being uniform strains, such as those which can be selectively propagated after serial experimental passages^46,52–54^. In this scenario, TE-based categories may not represent distinct strains, but they could rather reflect the presence of different proportions of the same sub-strains, whose effect on TE is amplified by the selective propagation of specific sub-strains in the different recipient rodent models. It can be hypothesized that isolates from categories 2-to-4 contained mixtures of different proportions of 19 K, supposedly CH1641, and one or more 21K-associated strains. Accordingly, the tg-shVRQ and tg-bov datasets explained most of the variability among categories 2-to-4, as their exclusion from the TE matrix strongly weakened the differences among categories 2-to-4 (Supplementary Fig. S5). Thus, tg-shVRQ and tg-bov would have always propagated 19 K because of their preferential selectivity for this strain component, either due to low susceptibility to 21 K or to high susceptibility to 19 K; but they did so with different TEs according to the quantity of 19 K in the original isolates. Conversely, rodent models less prone to select 19 K, such as tg-shARQ, tg-gtARQ and Bv109M, mainly propagated 21 K, accompanied by 19 K only when present in the isolates at high levels (i.e. isolates with high TE in tg-bov). In agreement with this interpretation, which implies that 21 K and 19 K are distinct sub-strains pre-existing as a mixture in the original isolates, rodent models less prone to select 19 K propagated 21 K even when inoculated with the 19 K UKB2 isolate, implying that this CH1641-like isolate also contained a 21 K component. Furthermore, individual Bv109M and tg-shARQ mice inoculated with the same scrapie isolate, alternatively propagated 19 K or 21 K, and the UKA2 and UKB2 isolates even induced the propagation of 19 K and 21 K a mixture in tg-gtARQ. Again, this implies that 19 K and 21 K should be considered discrete components, further corroborating our interpretation.

Finally, it is worth to highlight that even after the exclusion of the tg-shVRQ and tg-bov datasets from the TE matrix, the TE profile of category 1 remains distinguishable from all the others (Supplementary Fig. S5). This finding strongly suggests that isolates in category 1, besides being apparently devoid of the the 19 K sub-strain, have distinct biological properties and might even contain one single strain component. Thus, the simplest explanation for our data implies the presence of at least 2 different scrapie strains with different geographical distribution, one of which endows variable proportions of a supposedly CH1641 19 K component.

### Investigation of a UK goat herd suggests CH1641 as a hidden strain component in classical scrapie

Notably, the above-mentioned hypothesis would also explain the reason for the partial variability in TE profiles observed between goats deriving from the same herd (see N1 and N3 in Fig. 1), as it postulates that sub-strain components might coexist in different proportions in individual isolates. To get further insight into this phenomenon in goats exposed to a shared source of infectivity, we extended our studies towards a thoroughly investigated scrapie outbreak in a UK goat herd, from which the CH1641-like UKB2 and the classical scrapie UKA2 were sampled. In this herd, UKB2 was the only goat out of 71 tested which showed 19 K PrP^Sc^ in the brain; interestingly, all goats had a classical scrapie pattern in lymph nodes (LN), including the UKB2^28^.

As shown before, the 19 K UKB2 and the 21 K UKA2 brain isolates were both able to induce either 19 K or 21 K PrP^Sc^ in individual Bv109M (Fig. 5). Interestingly, 19 K was detected as the main PrP^Sc^ type in 7 out of 9 voles infected with UKB2, but only in 1 out of 15 of those infected with UKA2. Similarly, 7 out of 7 tg-gtARQ mice infected with UKB2 propagated a 19 K component, while only 3 out of 5 of those infected with UKA2 did so (see Supplementary Results). These findings suggest that the proportion of individual rodents propagating 19 K might reflect the presence of different proportions of 19 K in the original isolates. We thus inoculated Bv109M with the brain isolates from 2 additional scrapie infected goats from this herd, and with LN isolates from all 4 goats.

Overall, 3 brain (UKA2, UKB2, UKC2) and 2 LN (UKA2, UKC2) isolates gave positive transmissions in Bv109M, possibly reflecting low PrP^Sc^ levels in the pre-clinical donor goats UKB2 and UKD2 (Table 5). The only brain isolate which did not transmit in Bv109M was UKD2 that gave poor or negative transmissions also in the other rodent models, consistent with a very low infectivity titer (Supplementary Table S1). The other 3 brain isolates induced 19 K in Bv109M, although at different levels, ranging from 7% and 50% of positive voles for the two classical scrapie isolates (UKA2, UKC2), and to 78% for the CH1641-like isolate (UKB2). Importantly, one LN isolate (UKC2) induced 19 K too, but did so at a lower level than the respective brain isolate (9% *vs* 50%). Overall, only 8% positive Bv109M propagated 19 K upon inoculation with LN isolates, while 35% did so with brain isolates.

**Table 5:** Transmission in Bv109M of brain and LN TSE isolates from scrapie-positive goats deriving from the same UK goat herd.

ID	Clinical scrapie	Tissue	PrP^Sc^ in the inoculum	Attack rate	Survival time (days ± SD)	21 K PrP^Sc^ (n° voles)	19 K PrP^Sc^ (n° voles)
UKA2	yes	Brain	21 K	17/18	255 ± 86	14	1
		LN	21 K	2/5	302–638	2	
UKC2	yes	Brain	21 K	2/9	760; 760	1	1
		LN	21 K	11/12	421 ± 197	10	1
UKB2	no	Brain	19 K	9/11	481 ± 108	2	7
		LN	21 K	0/7			
UKD2	no	Brain	21 K	0/11			
		LN	21 K	0/6			

Brain and LN isolates from 3 of these goats were also inoculated in wild type mouse lines at APHA, in order to discriminate the unusual CH1641-like case from BSE. In these experiments, strains have been identified by their PrP^Sc^ deposition patterns in recipient mice, a method previously shown to allow the detection of BSE in goats and its discrimination from classical scrapie after a single passage in mice^5,55^. All isolates transmitted in all wild type mouse lines inoculated, with the exception of LN isolates from UKD2 and UKB2 (Table 6), thus confirming their low infectious titre. Transmissions to mice invariably resulted in the propagation of classical scrapie, which is in line with findings in wild type mice inoculated with isolates from this same herd^35^ (Table 3) and with CH1641-like isolates from sheep^53,56^.

**Table 6:** Transmission in wild type mouse lines of brain and lymphoid TSE isolates from scrapie-positive goats deriving from the same UK goat herd.

ID	Tissue	Mouse line	Attack rate	Survival time (days ± SD)	Strain*
UKA2	Brain (Obex)	C57	14/14	438 ± 20	Clas. Scrapie
		VM	15/15	542 ± 59	Clas. Scrapie
	LN	C57	20/20	514 ± 62	Clas. Scrapie
		VM	18/18	539 ± 65	Clas. Scrapie
UKB2	Brain (Frontal cortex)	C57	10/16	744 ± 144	Clas. Scrapie
		RIII	10/15	656 ± 171	Clas. Scrapie
		VM	5/15	685 ± 137	Clas. Scrapie
	Brain (Obex)	C57	19/20	516 ± 49	Clas. Scrapie
		RIII	10/11	436 ± 110	Clas. Scrapie
		VM	18/18	566 ± 61	Clas. Scrapie
	LN	C57	5/10	660 ± 51	Clas. Scrapie
		RIII	3/8	485 ± 75	Clas. Scrapie
		VM	0/16		Clas. Scrapie
UKD2	Brain (Obex)	C57	6/11	655 ± 42	Clas. Scrapie
		VM	4/13	620 ± 77	Clas. Scrapie
	LN	C57	0/14		Clas. Scrapie
		VM	0/15		Clas. Scrapie

*Strain identification was based on IHC as described elsewhere^5,55.^

This finding can be explained by the results obtained by discriminatory IHC of the donor goat brain. In doing so it should be recalled that an intraneuronal PrP^Sc^ accumulation detectable not only by C-terminal but also by N-terminal antibodies is indicative for classical scrapie^22^. This pattern was indeed detected in the UKB2 brain and was mainly seen in the obex region, the brain area from which the original UKB2 isolate was obtained, while the neurons of the frontal cortex showed a strong CH1641-like deposition (Supplementary Fig. S6). These differences are reflected in the wild-type mice bioassay. Indeed, although both regions produced classical scrapie, the sample from the frontal cortex was much less efficient in all experiments (Table 6). As wild type mice appear unable to replicate the 19 K CH1641-like component, these findings suggest that the lower transmission efficiency of the frontal cortex isolate in mice reflects the lower titre of the 21 K component in the frontal cortex compared to the obex, in agreement with discriminatory IHC findings in the same brain areas.

Overall, the findings obtained with goat isolates from this herd are in agreement and support our previous conclusion that 19 K and 21 K represent sub-strain components pre-existing in the prion isolates. We have also shown that 19 K and 21 K accumulate at variable levels and proportions in different goats, in different tissues, i.e. LN vs brain, and even in different brain areas of goats exposed to a common scrapie source. In principle, these observations might be explained by postulating a supposedly rare case of co-infection with 2 different scrapie strains, 21 K and 19 K. However, this seems unlikely as the 19 K/21 K divergence was observed in 16 out of 31 classical scrapie cases deriving from 5 different EU countries (Supplementary Table S1). This situation is similar to that observed in sheep scrapie, where the 19 K/21 K phenotype divergence has been observed with 18 out of 20 geographically unrelated isolates, thus suggesting an ontogenic link, rather than a simple strain mixing^46^. Importantly, we have also shown that the presence of 19 K depends on the biological properties of the isolates and relates to their geographical origin. Thus, we postulate that 19 K is a standard component of one or more, but not all, 21 K classical scrapie strains circulating in European goats. As in the vast majority of cases the dominant phenotype in the natural host is 21 K, 19 K is mostly present as a minor “hidden” component, which can be only recognized after selective propagation in susceptible hosts, such as tg-bov, tg-shVRQ and, to a lesser extent, Bv109M and tg-mice expressing wild type goat/sheep PrP. In this sense, 19 K might be continuously generated and selected against during scrapie replication in goat tissues, but positively selected during replication in specific rodent models. However, in rare cases 19 K can be positively selected even in its natural host, as shown by the variable presence in individual goats. The observed neurotropism of 19 K relative to 21 K is in line with data showing that the positive selection of 19 K in transgenic mice depends on high expression levels of PrP^C^^46^, and might explain its rare occurrence as the dominant phenotype in the brain of some scrapie affected sheep and goats.

Our conclusions that 19 K is a “hidden” component of a classical scrapie strain allow to reconcile some conflicting data that have been obtained in the past, by postulating that experimental CH1641 and 21 K classical scrapie are actually two sides of the same coin. CH1641 is an experimental scrapie strain that was originally isolated from a British scrapie case and propagated by serial transmission in sheep. Our results may suggest that serial intracerebral transmission in sheep led to positive selection of 19 K over 21 K in CH1641, given the neurotropism of 19 K and evidences that experimental transmission *per se* could mediate the emergence of CH1641-like properties in recipient sheep^46,57^. Interestingly, it has been shown that the PrP^Sc^ type in natural CH1641-like isolates does not fully match experimental CH1641, but shows intermediate properties between classical scrapie and experimental CH1641 suggesting the co-existence of 19 K and 21 K in a mixture^27^. In keeping with these data, 21 K has been selectively propagated in wild type mice inoculated with natural CH1641-like isolates, as shown here and in unrelated experiments^35,53,56^ while it has been historically difficult to infect wild type mice with experimental CH1641 isolates^20^. Thus, experimental CH1641 would represent an artificially selected extreme phenotype of a circulating classical scrapie strain, while the rare CH1641-like natural cases, which still endow significant levels of 21 K as visible in rodent bioassays, would represent the most deviant phenotypes in nature.

## Conclusions

The conclusions of this work are both, of practical relevance for improving EU surveillance toward a better identification of zoonotic prion strains, and of general relevance for understanding the biological properties of natural TSE isolates. Our study provides a range of rodent models for goat TSEs and BSE discrimination, as well as baseline data from a wide range of goat isolates, both available to scientists for future studies. Most importantly, our findings show that the goat BSE sample here studied was reliably discriminated from a wide range of biologically and geographically diverse goat TSE isolates. The identification of BSE could already be attained after a single passage in rodents, based on the relative transmission efficiency in different rodent models and on the PrP^Sc^ types associated. Previous studies showed overlapping disease phenotypes in goats with different PrP genotypes and genetic backgrounds challenged with BSE^33,58^, suggesting a low strain variation for BSE in goats and implying that our findings with a single BSE source are well representative of BSE in goats. Thus, our dataset provides the basis for building a reliable and practical approach in terms of discriminatory power and timing of the outcome for discriminating BSE cases from non-zoonotic TSEs in goat. To this purpose, a combination of 3 rodent models, tg-gtARQ or tg-shARQ, tg-shVRQ and tg-bov mice, seems to best fulfil the above criteria (Fig. 2).

In this work, we investigated the biological properties of natural TSE isolates by introducing the relative transmission efficiency of TSE isolates across several models, i.e. the TE profiles, as a biological signature of the isolates. This, coupled to PrP^Sc^ typing in recipient hosts, allowed to investigate the phenomenon of scrapie strains from a new perspective, i.e. the biological properties of TSE isolates were derived by their specific interaction with different recipient PrP species rather than by isolating rodent-adapted strains by serial passage in a single model. Rodent-adapted strains may stem from the selective amplification of some original strain component in the new host^54^, resulting in an incomplete representation of the original biological properties. In contrast, the approach used here may better represent the biological variability within each isolate, as it “reads” its composition through sieves with different mesh sizes, i.e. the various rodent models representing different selective environments. Indeed, thanks to the 19 K molecular signature which allowed to recognize the suggested CH1641 component, we unexpectedly found that a considerable number of classical scrapie isolates from European goats contains a mixture of at least two scrapie strain components or sub-strains, 19 K and 21 K, which co-exist in variable proportions in different individual goats and tissues and can be selectively propagated as discrete components in rodent models. It is conceivable that this phenomenon extends beyond the 19 K/21 K dichotomy currently detectable by WB, which would suggest that even isolates from which 19 K cannot be derived, such as those in category 1, could be actually composed of a mixture of PrP^Sc^ conformers. Detailed histopathological and immunohistochemical strain specific profiles of the ongoing subpassages in rodents may shed more light into it. We conclude that scrapie isolates have strain properties both, composite (i.e. they are mixtures of sub-strains which can be found in different proportions) and discrete (i.e. sub-strains can be isolated as distinct components in rodents), where it could be envisaged that phenomena such as competition or cooperation among sub-strains in different replication environments might play important roles in defining the biological properties of scrapie isolates and their evolution. Such a scenario is in line with the natural history of scrapie being a contagious disease, able to infect individuals with polymorphic PrP sequences and to colonize different tissues and cell types. All these factors represent changes in the replication environment and are likely to drive the emergence of PrP^Sc^ conformational variants which are under constant selective pressure as a mixture^46,59,60^. Our observation that the variation of TE profiles is partially explained by geographical proximity is in agreement with this scenario.

Finally, this conclusion has obvious impact on the investigation of the zoonotic potential of scrapie, as it is conceivable that the presence and proportion of specific sub-strains could drive the ability of scrapie isolates to cross species barriers. Interestingly, a subset of scrapie isolates studied here was able to adapt to different PrP sequences similarly or even better than BSE.

## Materials and Methods

### Ethics statement

All experimental protocols were approved by the authors’ respective institutions and national competent authorities and all procedures were carried out following ethical review in the authors’ respective institutions and in accordance with European Council directives 86/609 and 2010/63, as well as in compliance with the respective national legislations. For the experiments at WBVR, the Netherlands: Dutch Central Authority for Scientific Procedures on Animals, permit number AVD401002016522. At Friedrich-Loeffler-Institute, Germany: the competent authority of the Federal State of Mecklenburg-Western Pommerania, reference number LALLF 7221.3-2.1-012/03 and 7221.3-2.1-027/02. At APHA, United Kingdom: Animal & Scientific Procedures Act, 1986, Home Office License Number 70/6310. At INRA, France: supervision by Institut national de la recherche agronomique ethical committee (agreement numbers 02-032-02 for animal care facilities, 92–189 for animal experimentation). At INIA, Spain: Committee on the Ethics of Animal Experiments of the Instituto Nacional de Investigación y Tecnología Agraria y Alimentaria and the General Directorate of Madrid Community Government (Permit Numbers: CEEA 2009/003, CEEA 2011/050, PROEX 263/15). At ISS, Italy: Italian Legislative Decree 116/92 and then 26/2014; protocol approval and supervision by the Service for Biotechnology and Animal Welfare of the Istituto Superiore di Sanità, authorisation by the Italian Ministry of Health (decree number 84/12.B).

### TSE isolates from goats

With the aim to characterize the highest biological variability, natural TSE cases were selected in order to encompass the geographical, pathological and genetic variability observed in the field; details on the country of origin, the PrP genotype and the PrP^Sc^ type of the selected TSE isolates are reported in Table 1. In doing so, the panel of isolates included 30 brain tissues from naturally TSE-affected goats, along with one isolate from a goat experimentally infected with BSE and two isolates from goats experimentally infected with sheep or goat scrapie (Table 1). Only the three Dutch and the four UK brain samples were derived from animals from single holdings, all the others from different farms.

The goat brain samples had initially been found TSE-positive in the EU active surveillance plan with approved screening tests for the detection of TSEs and subsequent scrapie-BSE discriminatory analyses. A detailed analysis by Western blotting (WB) of PrP^Sc^ features of the inocula will be reported elsewhere^33^. One Italian goat (I15, Table 1) with Nor98/atypical scrapie has already been published before^40^.

Fifty percent brain macerates in water were prepared under sterile conditions in a microbial safety cabinet with sterile plastic and glass materials as follows. Materials, paper tissues and gloves were changed and the cabinet cleaned between each individual brain sample treatment. Samples were weighed, immersed in an equal part of water, minced with a scalpel blade, and left for 18 hr at 4 °C. Material was ground in a Pyrex glass Dounce and disrupted up to a homogenous paste. Completer homogenisation was obtained by forcing the suspension several times in a ten mL syringe through a 19 G needle. The 50% macerate was aliquoted in 1.5 mL tubes and stored at −80 °C.

### Rodent models

The rodent models used in this study are reported in Table 2; most of these models have been previously published and employed for characterizing sheep and goat scrapie^14,18,19,31,38,41,42,61–64^. All transgenic mouse lines are on PrP null background and are homozygous for the transgene.

In this study, different transgenic mouse lines have been used in order to model small ruminant PrP, expressing either wild type goat PrP (tg501), wild type sheep PrP (tgShpIX) and the most susceptible sheep PrP polymorphic variant (tg338) with the valine to alanine substitution at codon 136 (VRQ, as opposed to the ARQ wild type sequence). Tg501 mouse line expressing the goat wild type PrP^C^ in homozygosis was obtained by interbreeding the previously described hemizygous tg501 mouse line expressing one fold the PrP^C^ levels in the goat brain^62^. This lead to homozygosis for the goat PrP transgene within a murine PrP background (PrP mu^−/−^ go^+/+^). As expected, PrP^C^ expression levels determined in brain from homozygous tg501 animals was about 2-fold higher than PrP^C^ levels in goat brain as determined by dilution experiments in WB (data not shown).

Of note, although sheep and goat have different PRNP nucleotide sequences, they share the same PrP amino acid sequence. Hence, the transgenic mouse line used here to model wild type (ARQ/ARQ) sheep PrP, tgShp IX, share the same PrP amino acid sequence with tg501. However, the genetic background is different (B6CBAx129Ola in tgshp IX and FVB in tg501) as is the PrP expression level with 4–8x in tgShp IX and 2x in tg501 as compared to sheep/goat brain respectively. These two lines will be referred to as tg-shARQ and tg-gtARQ in order to emphasize their similarity in terms of PrP sequence.

### Bioassay

Aliquots of the TSE isolates were dispatched to all participating laboratories as 50% macerates. Brain macerates were adjusted to 10% (w/v) in phosphate buffered saline (PBS) or 5% glucose. Groups of six-to-eight weeks old rodents were anesthetized and inoculated intracerebrally with 20 μl of brain homogenate. Animals were examined twice a week until neurological signs appeared, after which they were examined daily. Animals were culled with carbon dioxide before neurological impairment was such as to compromise their welfare, in particular their ability to drink and feed adequately, or at the set end point of the experiment. At post-mortem, brains from inoculated mice were removed and divided sagittally. Half brain was frozen for WB analysis of PrP^Sc^ and half fixed in formol-saline for histological analysis. The attack rate was calculated as the number of animals scoring positive at post-mortem/number inoculated. Animals found dead or culled for intercurrent disease before 100 days post-inoculation (dpi) and scoring negative at post-mortem were excluded from analyses. The survival time for animals scoring positive at post-mortem was calculated as the time from inoculation to culling or death.

In order to analyse and compare the transmission efficiency of isolates among the various rodent models, we introduced a new parameter, called transmission efficiency (TE), which takes into consideration both the attack rate and the survival time. To do so, mean survival times were categorized into 6 categories according to the observed overall variation (see S1 Table): those with the shortest mean survival time (<200 dpi) were assigned the highest TE value (ST = 1); all others experiments having mean survival times >200 dpi were divided into 5 categories by increasing steps of 100 dpi. Then, equally spaced ST values were assigned to these categories by decreasing TE of 0,17 for each step (which represent 1 divided by the number of categories). In summary, each category was assigned to a survival time value (ST) between 0 and 1, as follow: 1,00 for survival times <200 dpi; 0,83 for survival times between 200 and 299 dpi; 0,67 for survival times between 300 and 399 dpi; 0,50 for survival times between 400 and 499 dpi; 0,33 for survival times between 500 and 599 dpi; 0,17 for survival times >600 dpi. TE was then calculated by multiplying the attack rate and ST values.

### Molecular analysis of PrP^Sc^ in rodent models by western blotting

In all rodent models the detection and typing of PrP^Sc^ was performed by WB analysis of proteinase K-treated PrP^Sc^ in brain homogenates. Each laboratory used its own WB method and typing strategy, which are described in detail in Supplementary Methods.

### Pathology and Immunohistochemistry

At APHA, wild type mice (C57/BL6 and VM) were challenged with brain tissue or mesenteric lymph node from three of the four UK isolates (G08-1469, G08-1475, G08-1446). In these experiments, TSE diagnosis was based on the detection of PrP^Sc^ using IHC.

Fixed brains were cut at 4 different coronal levels to reveal frontal cortex, hippocampus with thalamus, midbrain, and brain stem with cerebellum. Histological sections from the above levels were prepared and stained either with H&E to assess spongiosis or were subjected to immunohistochemistry using the polyclonal antibody Rb486 as described^65^.

Discriminatory IHC in goat tissues was performed according to published protocols^4^.

### Definitions and nomenclature

We will use PrP^Sc^ as a general term for indicating pathological aggregates of misfolded PrP; the term PrP^res^ will be used specifically to refer to the protease-resistant core of PrP^Sc^.

Different types of PrP^Sc^ are discriminable by molecular methods, yielding different PrP^res^ cores. In WB, PrP^res^ appears usually as three protein bands with apparent molecular masses between 17 and 30 kDa; the top, middle and lower one are respectively diglycosylated, monoglycosylated and non-glycosylated forms of the same amino acid sequence. An exception is atypical/Nor98 scrapie which has variably processed PrP^res^ bands, of which a low band between 7–9 kDa is a major and characteristic fragment. In this manuscript, PrP^Sc^ types are named by the apparent molecular mass (MM) of the unglycosylated PrP^res^ fragment, i.e. 21 K or 19 K.

The apparent MM of the unglycosylated PrP^res^ fragments varied with the different WB methods used here, owing to the different gels and molecular markers used. For example, classical scrapie and BSE were measured as 21 kDa and 20 kDa, respectively, in RIII mice; as 19 kDa and 18 kDa in tg-shARQ, tg-goat and tg-bov; and as 18 kDa and 17 kDa in Bv109M. To address this issue, and considering that scrapie and BSE have been historically mostly referred as 21 kDa and 19 kDa, we introduced a common nomenclature to refer to the different PrP^Sc^ types as follows: 19 K for BSE -like PrP^Sc^; 21 K for scrapie-like PrP^Sc^ and 8 K for Nor98-like PrP^Sc^.

## Supplementary information

Supplementary information.

Supplementary Table S1.

## Data Availability

All relevant data are within the manuscript and its Supporting Information files.
