# Supplementary material for: Characterization of goat prions demonstrates geographical variation of scrapie strains in Europe and reveals the composite nature of prion strains
Source: Sci Rep. 2020 Jan 8;10:19. doi: 10.1038/s41598-019-57005-6 (PMC6949283; doi:10.1038/s41598-019-57005-6)
Supplement: Supplementary file 1 — Supplementary information. [file 41598_2019_57005_MOESM1_ESM.pdf]

SUPPLEMENTARY INFORMATION

Characterization of goat prions demonstrates geographical variation of scrapie strains in Europe and reveals the composite nature of prion strains

Romolo Nonno[1], Alba Marin-Moreno[2], Juan Carlos Espinosa[2], Christine Fast[3], Lucien Van Keulen[4], John Spiropoulos[5], Isabelle Lantier[6], Olivier Andreoletti[7], Laura Pirisinu[1], Michele A. Di Bari[1], Patricia Aguilar-Calvo[2], Theodoros Sklaviadis[8], Penelope Papasavva‑Stylianou[9], Pier Luigi Acutis[10], Cristina Acin[11], Alex Bossers[4], Jorge G. Jacobs[4], Gabriele Vaccari[1], Claudia D'Agostino[1], Barbara Chiappini[1], Frederic Lantier[6], Martin H. Groschup[3], Umberto Agrimi[1], Juan Maria Torres[2], Jan P.M. Langeveld[4]*

[1] Istituto Superiore di Sanità, Department of Food Safety, Nutrition and Veterinary Public Health, Rome, Italy

[2] Centro de Investigación en Sanidad Animal, CISA-INIA, Madrid, Spain

[3] Institute of Novel and Emerging Infectious Diseases, Friedrich-Loeffler-Institute, Greifswald-Isle of Riems, Germany

[4] Wageningen BioVeterinary Research, Lelystad, the Netherlands

[5] Animal and Plant Health Agency, New Haw, Addlestone, Surrey, United Kingdom

[6] INRA-Centre Val de Loire, Infectiologie et Santé Publique, Nouzilly, France

[7] UMR INRA ENVT 1225- IHAP, École Nationale Vétérinaire de Toulouse, Toulouse, France

[8] Laboratory of Pharmacology, School of Health Sciences, Department of Pharmacy, Aristotle University of Thessaloniki, Thessaloniki, Greece

[9] Veterinary Services, Nicosia, Cyprus

[10] Istituto Zooprofilattico Sperimentale del Piemonte, Liguria e Valle d'Aosta, Torino, Italy

[11] Centro de Encefalopatías y Enfermedades Transmisibles Emergentes, Facultad de Veterinaria, Universidad de Zaragoza, Zaragoza, Spain

* **Corresponding author**
Email: romolo.nonno@iss.it

Supplementary information:

- **Supplementary Methods** **p. 2**
- **Supplementary Results** **p. 4**
- **Supplementary References** **p. 8**
- **Supplementary Figs S1-S6** **p. 10**

Supplementary Methods

Molecular analysis of PrP^{Sc} in rodent models by western blotting

Antibodies. Purified PrP-specific monoclonal antibodies (mAbs) were: 12B2 and 9A2 (WBVR, Lelystad, Netherlands), Sha31 and SAF84 (SpiBio, France), L42 and P4 (R-Biopharm, Germany). The mapped amino acid sequences (goat PrP numbering) of these mAbs are: 93WGQGG97 for 12B2; 102WNK104 for 9A2; 148YEDRYYRE155 for Sha31; 167YRPVDQY172 for SAF84; 148YEDRYY153 for L42 and 93WGQGGSH99 for P4 using Pepscan analysis [1-3]. Based on their respective epitopes, these antibodies can be grouped into 3 categories: i) 12B2 and P4 recognize overlapping epitopes at the N-terminus of PrP^{res} which is differentially cleaved by PK in scrapie, BSE, and CH1641, and are thus useful for discrimination. The 19K fragment and its two glycosylated forms are not recognized by 12B2 and P4. The advantage of 12B2 is that it is not dependent on species differences while P4 has significantly reduced affinity for bovine, murine and bank vole PrP [4,5]; ii) 9A2, Sha31 and L42 recognize epitopes in the core of PrP^{res}; and iii) SAF84 recognizes a more C-terminal epitope and is able to detect not only the main C-terminal PrP^{res} fragment, but also a shorter C-terminal fragment of around 10-14 kDa and its two glycosylated forms not detected by mAbs in the previous groups; this antibody helps to characterize the PrP^{Sc} types in ovine CH1641 scrapie or bovine H-BSE [4,6].

Tg-gtARQ and tg-bov mice. Frozen brain tissues (175±20 mg) were homogenized in 5% glucose in distilled water in grinding tubes (Bio-Rad) adjusted to 10% (w/v) using a TeSeETM Precess 48TM homogenizer (Bio-Rad) following manufacturer's instructions. Presence of PrP^{res} in transgenic mouse brains was determined by WB, using the reagents of the ELISA commercial test TeSeE (Bio-Rad). Brain homogenates (10-100 µl of a 10% (w/v)) were prepared following previously described protocol [7,8] and samples were loaded in 12% Bis-Tris Gel (Criterion XT, Bio-Rad). Proteins were electrophoretically transferred onto PVDF membranes (Millipore) which were blocked O/N with 2% BSA blocking buffer. For immunoblotting, membranes were incubated with Sha31 or 12B2 mAbs at a concentration of 1 µg/mL. Immunocomplexes were detected by incubating the membranes for 1 hour with horseradish peroxidase conjugated anti mouse IgG (GE Healthcare Amersham Biosciences). Immunoblots were developed with enhanced chemiluminiscence ECL Select (GE Healthcare Amersham Biosciences). Images were captured using ChemiDocTM WRS+ System and processed using Image Lab 5.2.1 Software.

Tg-shARQ mice. A specific precipitation by PTA was performed, followed by using Western blot analysis as previously described [9,10]. A 10% (w/v) brain homogenates was prepared in 0.42 mmol/L sucrose solution, containing 0.5% deoxycholic acid sodium salt and 0.5% Nonidet P-40 by using a Ribolyser (Hybaid, Heidelberg, Germany). Gross cellular debris was removed by centrifugation at 6000 rpm for 5 minutes at room temperature. A 200 µl aliquot of the supernatant was incubated with proteinase K to a final

concentration of 50 µg/ml proteinase K and incubated at 55°C for 1 hour. Digestion was terminated by addition of 4µl of Pefabloc (Roche, Mannheim, Germany) and heating for 5 minutes at 95°C. Digested homogenates were mixed with PTA to a final concentration in the sample of 0.3% (w/v) PTA. Samples were incubated at 37°C for 60 minutes with constant agitation before centrifugation at 13,300 rpm for 30 minutes at room temperature. After careful removal of the supernatant, pellets were resuspended in sample buffer and heated for 5 minutes at 95°C. After a short centrifugation, samples were loaded on 16% Tris-polyacrylamide gels and subjected to SDS-PAGE. Gels, were transferred onto polyvinylidene fluoride membrane (Millipore, Billerica, MA) and blocked for 1 hour in 5% (w/v) nonfat milk powder in PBS containing 0.1% (v/v) Tween-20 (PBST). PrP^{Sc} bound to the membrane was detected using the mAb L42 (R-Biopharm, Darmstadt, Germany) and mAb P4 (R-Biopharm, Darmstadt, Germany) respectively both at a concentration of 0.4_µg/ml, which was incubated on the membranes for 1 hour at room temperature. The membranes were washed three times with PBS-Tween (PBST) and incubated in a 0.15 µg/ml concentration of alkaline phosphatase–conjugated anti-mouse Ig (Dianova, Hamburg, Germany) in PBST for 1 hour at room temperature. The membranes were finally washed three times with PBST, and the bound antibodies were detected using the chemiluminescent substrate CDP Star (Tropix, Bedford, MA) and direct visualization in an image analysis system (Versa Doc, Quantity One; Bio-Rad, Munich, Germany).

Tg-shVRQ and tga20 mice. Frozen brain tissue (175±20 mg) was homogenized in 5% glucose in distilled water in grinding tubes (Bio-Rad) adjusted to 10% (w/v) using a TeSeETM Precess 48 homogenizer (Bio-Rad). A western blot kit (TeSeE WB kit Bio-rad) was used following the manufacturer's recommendations and PrP^{res} detection used a mAb Sha31 at a dilution of 1/8,000.

RIII mice. Longitudinally cut mouse brain halves were homogenized to ten percent tissue homogenates using a polypropylene pestle (Sigma-Aldrich Z359947-100EA), 1.5 mL Eppendorf polypropylene vials, and a pellet pestle cordless motor (Kontes K749540-0000). The Triplex-WB procedures have been described in detail elsewhere [3], using mAbs 12B2, Sha31, and SAF84 at respective concentrations of 0.2, 0.1 and 0.5 µg IgG ml^{-1}. For goat and mouse brain samples, amount of PrP^{res} signals in ng per mg brain tissue were calculated from the mAb Sha31 signal using as for normalisation reference in each gel a known quantity of rec-ovine PrP-ARQ (source: Rezai, INRA, Jouy en Jozasse, France). Each brain sample was analysed at least three times on different days. Statistical analyses of PrP^{res} triplex-WB analytical data were performed using GraphPadPrism from GraphPad Software Inc.,USA.

Bv109M voles. Brain homogenates (20% w/v) were prepared as previously described [11]. After adding an equal volume of 100 mM of Tris–HCl containing 4% sarkosyl, the homogenates were incubated for 30 min at 37 °C with gentle shaking. PK (Sigma-Aldrich) was added at a fnal concentration of 200 µg/ml and then the samples were incubated for 1 h at 55 °C with gentle shaking. Protease treatment was stopped with 3 mM of PMSF (Sigma-Aldrich). Aliquots of samples were added with an equal volume of isopropanol/butanol

(1:1 v/v) and centrifuged at 20,000g for 5 min. Supernatants were discarded and the pellets were resuspended in denaturing sample bufer and heated for 10 min at 90 °C. Electrophoresis and Western blotting were performed as previously described [11]. PrP^{res} was detected with mAbs SAF84 and 12B2. Following incubation with horseradish peroxidase-conjugated anti-mouse immunoglobulin (Pierce Biotechnology, Rockford, IL, USA) at 1:13,000, the PrP bands were detected by enhanced chemiluminescent substrate (SuperSignal Femto, Pierce, Rockford, IL, USA) and VersaDoc imaging system (Bio-Rad). The chemiluminescence signal was quantified by QuantityOne software (Bio-Rad). The SAF84/12B2 antibody ratio was used in order to discriminate BSE-like (referred to as 19K) from scrapie-like (referred to as 21K) PrP^{res}. The SAF84/12B2 absolute ratio was determined by calculating the chemiluminescence signal of the diglycosylated PrP^{res} band separately with SAF84 and 12B2 monoclonal antibodies. To obtain the relative SAF84/12B2 ratio, we calculated the absolute ratio of SAF84/12B2 volumes for each sample and for a scrapie sample used as reference and then divided the absolute ratio of each sample by the absolute ratio of the scrapie control. Samples resulting in a relative SAF84/12B2 ratio >2 were considered BSE-like (19K), while those with ratio <2 were considered scrapie-like (21K).

Supplementary Results

Molecular analysis of PrP^{Sc} in rodent models by western blotting

Tg-gtARQ and tg-bov mice. PrP^{Sc} in tg-gtARQ and tg-bov transgenic mice was determined by a WB method for ovine, caprine and bovine TSEs, able to discriminate the strain-specific PrP^{Sc} types in several transgenic mouse lines by mobility differences of the PrP^{res} bands detected by Sha31 [7,8,12]. With this method, we compared PrP^{Sc} in goat isolates and in recipient tg-goat mice (Fig 4A and 4B). In general, the WB type of goat isolates was mostly preserved upon transmission in tg-gtARQ. The goat-BSE isolate induced the typical PrP^{res} banding pattern, characterized by 19K fragment for the unglycosylated-band and prominent diglycosylated species (Fig 4A). This pattern was indistinguishable from that observed in hemizygous tg-gtARQ mice infected with the same goat-BSE used here, or with cattle-BSE isolate [13]. Atypical scrapie I15 faithfully propagated its biochemical signature, characterized by a ladder-like pattern and a low unglycosylated band of around 8 kDa [13] (Fig 4B). The CH1641-like UKB2 isolate showed a double 19-21K PrP^{res} unglycosylated band by this WB method, and preserved this signature upon transmission in tg-gtARQ (Fig 4A). Out of 23 classical scrapie isolates, 21 preserved the 21K PrP^{Sc} signature. However two of them, UKA2 and F14, gave rise to 19-21K double unglycosylated PrP^{res} similarly to UKB2, although 2 out of 5 mice infected with UKA2 exhibited a single 21K unglycosylated band (Fig 4A). Finally, in tg-gtARQ mice there was no overlap among PrP^{Sc} types induced by classical scrapie isolates or goat-BSE, as only goat-BSE resulted in pure 19K PrP^{res}, while classical scrapie isolates propagated as 21K or mixed 19-21K PrP^{Sc} types.

In contrast, in tg-bov mice a pure 19K PrP^{res} was propagated upon inoculation of several classical scrapie isolates (Fig 4C), as already observed in the same transgenic mouse line inoculated with sheep scrapie [14,15]. Indeed, 21K PrP^{res} was preserved only with 4 scrapie inocula (I2, I3, I9, F16), and in 1/6 mice inoculated with S2. All other tg-bov mice infected with scrapie isolates, including the CH1641-like UKB2 and 12 classical scrapie isolates, propagated 19K PrP^{Sc} (Fig 4C). Goat-BSE led to the propagation of a faithful 19K PrP^{res}, which could be differentiated from the scrapie-derived 19K by the mostly diglycosylated glycotype (Fig 4C), as previously reported [14,15].

Tg-shARQ mice. PrP^{Sc} in tg-shARQ mice was analysed by a discriminatory WB method which allows to determine the apparent MM of PrP^{res}, and also relies on the presence of the N-terminal P4 epitope, which after PK treatment is either removed or not to yield respectively 19K PrP^{Sc} (BSE-like), or 21K PrP^{Sc} (scrapie-like). This parameter was determined by the relative PrP^{res} signal of antibody P4 compared to that of PrP core-specific antibody L42, expressed by the P4/L42 signal ratio (Supplementary Fig. S2). All samples were analysed in comparison with classical scrapie controls. Individual samples having PrP^{res} >0,5 kDa lower than the scrapie control and with P4/L42 ratio <0,4 (i.e. relative absence of the N-terminal P4 epitope), were considered 19K PrP^{Sc}, while those with values close to the scrapie control were considered 21K PrP^{Sc}.

Atypical scrapie I15 faithfully propagated its 8K biochemical signature in tg-shARQ mice. Most classical scrapie isolates induced 21K PrP^{Sc} in tg-shARQ mice. However, in keeping with above findings in tg-gtARQ, individual mice with 19K PrP^{res} were detected after inoculation with few classical scrapie isolates and with the CH1641-like isolate UKB2. Of note, the proportion of mice with 19K PrP^{res} in groups inoculated with classical scrapie was very low (1 out of 13 in F6, 1 out of 9 in F14 and 3 out of 14 in S2), while it was higher in mice infected with UKB2 (3 out of 6). Tg-shARQ mice inoculated with goat BSE mostly showed 19K PrP^{res}; however, it was found that 2 out of 12 mice in this group had 21K PrP^{res}. Thus, even taking into consideration incubation times which were similar for goat-BSE and some of the classical scrapie isolates resulting in 19K PrP^{res} (Supplementary Table S1 and Table 3), reaching a definitive discrimination from BSE after a single passage in tg-shARQ might not be straightforward, and pathological assessments or second passages might be needed in some cases.

Tg-shVRQ and tga20 mice. PrP^{Sc} in tg-shVRQ and tga20 mice was determined by looking at the electrophoretic mobility and the glycosylation pattern of PrP^{res} as detected by mAb Sha31. In tg-shVRQ mice, the Nor98/atypical scrapie isolate I15 faithfully propagated its 8K biochemical signature, as previously shown with sheep Nor98 [16], and goat BSE preserved the 19K signature. Several classical scrapie isolates induced a faithful 21K PrP^{Sc} signature in tg-shVRQ mice, but the CH1641-like UKB2 isolate and 11 classical scrapie isolates resulted in 19K PrP^{Sc} (Supplementary Table S1). With this WB approach, the 19K PrP^{res} derived from scrapie cases in tg-shVRQ mice was not easily distinguishable from that derived from goat BSE in the same mice. Thus, although most scrapie cases resulting in 19K PrP^{res} transmitted more efficiently

than goat BSE (Supplementary Table S1 and Table 3), reaching a definitive discrimination from BSE after a single passage in tg-shVRQ might not be straightforward, and pathological assessments or second passages might be needed in some cases.

In contrast to the findings in tg-shVRQ, tga20 mice faithfully propagated 21K PrP^{Sc} from all classical scrapie cases, except UKB2 (Supplementary Table S1). In these mice, CH1641-like UKB2 isolate and goat BSE induced 19K PrP^{res}, with tga20 mice infected with UKB2 showing less glycosylated 19K PrP^{res} compared to those infected with goat BSE.

RIII-mice. PrP^{res} analyses of the RIII mouse brains were performed by triplex WB with antibodies 12B2, Sha31 and SAF84 using the same method previously used to characterize RIII mouse-adapted reference prion strains [3]), which allowed to confidently differentiate BSE/301C and four scrapie strains from each other. Four independent parameters for typing the isolates were used: (1) molecular mass of the nonglycosylated moiety as probed with core specific antibody Sha31, (2) the relative N-terminal 12B2 epitope content by calculating the 12B2/Sha31 signal ratio of the collective three PrP^{res} bands, (3) glycoprofile by calculating the ratio between monoglycosylated and diglycosylated PrP^{res} fraction (M/D) as revealed with antibody Sha31, and (4) the dual population marker as revealed by the ratios exceeding 1.2 for SAF84/Sha31 signals in the 24 kDa region, as it occurs in CH1641 scrapie and H-type BSE [2,3,17]. The dual population marker indicates co-occurrence of an additional triple band PrP^{res} population (PrP^{res}#2) with C-terminal PrP^{res} fragments which have molecular masses between 10-25 kDa, about 7-8 kDa shorter than the three bands between 17-32 kDa of PrP^{res}#1, with PrP^{res}#2 being not detected by mAb Sha31 [2]. All the data per isolate were usually obtained from up to three RIII mice (see Supplementary Fig. S2) since whole brains from most mice were fixed in paraformaldehyde for immunohistochemistry. Each analysis was performed three times in which it appeared that outcome were quite homogenous leading to overall SD's varying for kDa, 12B2/Sha31, M/D and SAF84/Sha31 at 24 kDa respectively ± 0.3 kDa, ± 0.12, ± 0.11, and ± 0.08. For two isolates, F2 and F6, no frozen brain material was available and for the S2 and S3 only one brain was available. One isolate from France - F16 - did produce in the mice too low PrP signals for correct molecular data. None of the Italian isolates tested including the AS/Nor98 case were clinically or WB positive. All other field isolates induced in RIII mice the classical scrapie signature, with an apparent MW of the non-glycosylated PrP band of 21 kDa, while it was 19 kDa in goat BSE (Supplementary Figs S2 and S3). Similarly, the content of N-terminus marker (12B2 epitope) was also of scrapie type, with 12B2/Sha31 ratios varying between 0.70-0.91, while that of goat BSE was below 0.1 (Supplementary Fig. S3A). The relation between the relative N-terminal 12B2 epitope content and the kDa values of the non-glycosylated fraction did correspond very well, which corroborates the concept that both parameters depends on N-terminal processing of PrP^{Sc} by proteinase K. The glycoprofile marker ratio's (M/D, Sha31) in the field isolates varied between 0.50-0.90. Also the reference 301C-BSE, 87A and ME7 have M/D values in this

range, in contrast to 22C and 79A that exhibit values are around 1 and higher (Supplementary Fig. S3A). For comparison M/D values in BSE of small ruminants are between 0.2-0.4 which is lower than for BSE and the scrapie strains in RIII mice. In CH1641 samples this ratio shows values between 0.4 and 0.5 [2,17]. None of the samples did exhibit an indication for the presence of a PrP^{res}#2 population since all field samples and goat BSE in RIII were close to 1 for the marker used (Supplementary Fig. S3B). Altogether by molecular analysis of the limited number of mice per isolate tested by WB, no indication of a BSE- or CH1641-like case in any of the field samples was evident. Indeed, all field isolates which gave positive transmission behaved as classical scrapie strains and, based on the glycoprofile (M/D ratios with Sha31), they were similar to 87A and/or ME7 strains. Finally, the RIII mice did not select any 19K component from scrapie as could at least have been expected to show up from a CH1641-like case as UK-B2.

Bv109M voles. PrP^{Sc} in Bv109M was analysed by a discriminatory WB method which relies on the presence of the N-terminal 12B2 epitope, which after PK treatment is either partially removed, or not, to yield respectively 19K PrP^{Sc} (BSE-like), or 21K PrP^{Sc} (scrapie-like). This parameter was determined by the SAF84/12B2 relative diglycosylated PrP^{res} signal (see methods). All samples were analysed in comparison with classical scrapie controls. Individual samples with SAF84/12B2 relative ratio >2 (i.e. relative absence of the N-terminal 12B2 epitope), were considered 19K PrP^{Sc}, while those with values <2 were considered 21K PrP^{Sc}. Furthermore, SAF84 allowed to detect the ~13 kDa C-terminal PrP^{res} fragment which characterizes some PrP^{Sc} type (Supplementary Fig. S4), such as that of CH1641 [6].

Bv109M voles inoculated with goat BSE showed either 19K PrP^{res} (n=4) or 21K PrP^{res} (n=4), in keeping with previous observations with sheep BSE and variant CJD in the same vole line [12]. Most classical scrapie isolates induced 21K PrP^{Sc} in Bv109M. However, in keeping with above findings in transgenic mice and previous findings in Bv109M inoculated with scrapie isolates [15], individual voles with 19K PrP^{res} were detected after inoculation with few classical scrapie isolates and with the CH1641-like isolate UKB2. Of note, the proportion of mice with 19K PrP^{res} in groups inoculated with classical scrapie was very low (2 out of 6 in F6, 2 out of 6 in S2, 1 out of 5 in S3 and 1 out of 15 in UKA2), while it was higher in mice infected with UKB2 (7 out of 9). The 19K PrP^{res} derived from these scrapie cases was frequently, but not invariably, accompanied by an additional 13 kDa C-terminal PrP^{res} fragment, reminiscent of that observed in sheep with CH1641 [6], which was absent in Bv109M infected with goat BSE (Supplementary Fig. S4). Due to the propagation of either 19K or 21K PrP^{Sc} in Bv109M infected with goat BSE and some scrapie isolates, a definitive BSE discrimination in Bv109M might require pathological assessments or second passages [12].

Supplementary References

1. Langeveld JP, Jacobs JG, Erkens JH, Bossers A, van Zijderveld FG, van Keulen LJ. Rapid and discriminatory diagnosis of scrapie and BSE in retro-pharyngeal lymph nodes of sheep. BMC Vet Res. 2006 Jun 9;2:19.
2. Jacobs JG, Sauer M, van Keulen LJ, Tang Y, Bossers A, Langeveld JP. Differentiation of ruminant transmissible spongiform encephalopathy isolate types, including bovine spongiform encephalopathy and CH1641 scrapie. J Gen Virol. 2011 Jan;92(Pt 1):222-32. doi: 10.1099/vir.0.026153-0
3. van Keulen LJ, Langeveld JP, Dolstra CH, Jacobs J, Bossers A, van Zijderveld FG. TSE strain differentiation in mice by immunohistochemical PrP(Sc) profiles and triplex Western blot. Neuropathol Appl Neurobiol. 2015 Oct;41(6):756-79.
4. Polak MP, Zmudzinski JF, Jacobs JG, Langeveld JP. Atypical status of bovine spongiform encephalopathy in Poland: a molecular typing study. Arch Virol. 2008;153(1):69-79. Epub 2007 Sep 26.
5. Langeveld JP, Erkens JH, Rammel I, Jacobs JG, Davidse A, van Zijderveld FG, et al. Four independent molecular prion protein parameters for discriminating new cases of C, L, and h bovine spongiform encephalopathy in cattle. J Clin Microbiol. 2011 Aug;49(8):3026-8.
6. Vulin J, Biacabe AG, Cazeau G, Calavas D, Baron T. Molecular typing of protease-resistant prion protein in transmissible spongiform encephalopathies of small ruminants, France, 2002-2009. Emerg Infect Dis. 2011 Jan;17(1):55-63. doi: 10.3201/eid1701.100891
7. Padilla D, Béringue V, Espinosa JC, Andreoletti O, Jaumain E, Reine F, et al.. Sheep and goat BSE propagate more efficiently than cattle BSE in human PrP transgenic mice. PLoS Pathog. 2011 Mar;7(3):e1001319. doi: 10.1371/journal.ppat.1001319
8. Aguilar-Calvo P, Espinosa JC, Andréoletti O, González L, Orge L, Juste R, et al. Goat K222-PrPC polymorphic variant does not provide resistance to atypical scrapie in transgenic mice. Vet Res. 2016 Sep 22;47(1):96.
9. Gretzschel A, Buschmann A, Eiden M, Ziegler U, Lühken G, Erhardt G, et al. Strain typing of German transmissible spongiform encephalopathies field cases in small ruminants by biochemical methods. J Vet Med B Infect Dis Vet Public Health. 2005 Mar;52(2):55-63.
10. Kaatz M, Fast C, Ziegler U, Balkema-Buschmann A, Hammerschmidt B, Keller M, et al. Spread of classic BSE prions from the gut via the peripheral nervous system to the brain. Am J Pathol. 2012 Aug;181(2):515-24. doi: 10.1016/j.ajpath.2012.05.001
11. Pirisinu L, Marcon S, Di Bari MA, D'Agostino C, Agrimi U, Nonno R. Biochemical characterization of prion strains in bank voles. Pathogens. 2013 Jul 2;2(3):446-56. doi: 10.3390/pathogens2030446

12. Espinosa JC, Nonno R, Di Bari M, Aguilar-Calvo P, Pirisinu L, Fernández-Borges N, et al. PrPC Governs Susceptibility to Prion Strains in Bank Vole, While Other Host Factors Modulate Strain Features. J Virol. 2016 Nov 14;90(23):10660-10669. doi: 10.1128/JVI.01592-16
13. Aguilar-Calvo P, Espinosa JC, Pintado B, Gutiérrez-Adán A, Alamillo E, Miranda A, et al. Role of the goat K222-PrP(C) polymorphic variant in prion infection resistance. J Virol. 2014 Mar;88(5):2670-6. doi: 10.1128/JVI.02074-13
14. Espinosa JC, Andréoletti O, Castilla J, Herva ME, Morales M, Alamillo E, et al. Sheep-passaged bovine spongiform encephalopathy agent exhibits altered pathobiological properties in bovine-PrP transgenic mice. J Virol. 2007 Jan;81(2):835-43.
15. Konold T, Nonno R, Spiropoulos J, Chaplin MJ, Stack MJ, Hawkins SA, et al. Further characterisation of transmissible spongiform encephalopathy phenotypes after inoculation of cattle with two temporally separated sources of sheep scrapie from Great Britain. BMC Res Notes. 2015 Jul 24;8:312. doi: 10.1186/s13104-015-1260-3
16. Le Dur A, Béringue V, Andréoletti O, Reine F, Laï TL, Baron T, et al. A newly identified type of scrapie agent can naturally infect sheep with resistant PrP genotypes. Proc Natl Acad Sci U S A. 2005 Nov 1;102(44):16031-6.
17. Langeveld JP, Jacobs JG, Erkens JH, Baron T, Andréoletti O, Yokoyama T, et al. Sheep prions with molecular properties intermediate between classical scrapie, BSE and CH1641-scrapie. Prion. 2014;8(4):296-305. doi: 10.4161/19336896.2014.983396

Supplementary Figure S1. Western blot analysis of PrP^{Sc} in tg-shARQ mice.

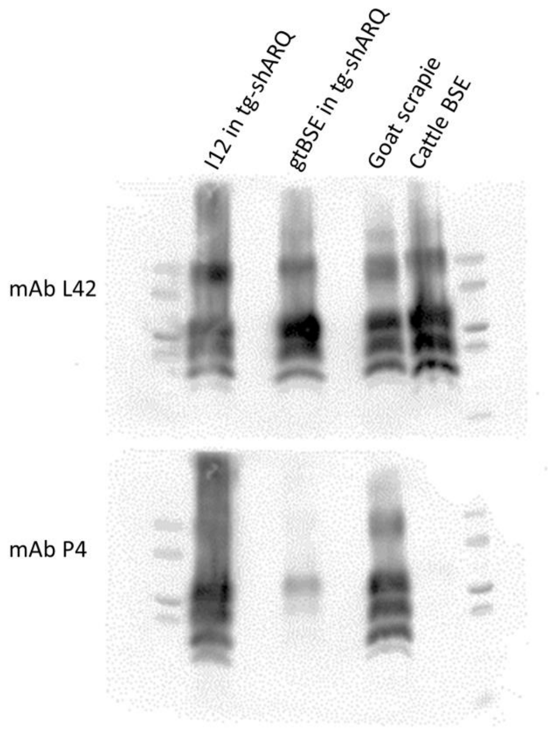

Representative discriminatory WB showing 21K/19K discrimination in tg-shARQ mice. Brain homogenates from tg-shARQ mice infected with the scrapie isolate I12 or with goat BSE were PK-treated and PTA-precipitated, along with brain homogenates from goat scrapie and cattle BSE used as controls. Replica blots were revealed with the core mAb L42 (upper panel) or with the N-terminal mAb P4 (lower panel), whose epitope is partially cleaved in 19K PrP^{res}. Note that the tg-shARQ sample with goat BSE shows a strongly decreased signal with P4 compared with L42, similarly to the cattle BSE control. In contrast, tg-shARQ and goat scrapie fully preserve the P4 epitope upon PK digestion.

Supplementary Figure S2. Western blot analysis of PrP^{Sc} in RIII mice.

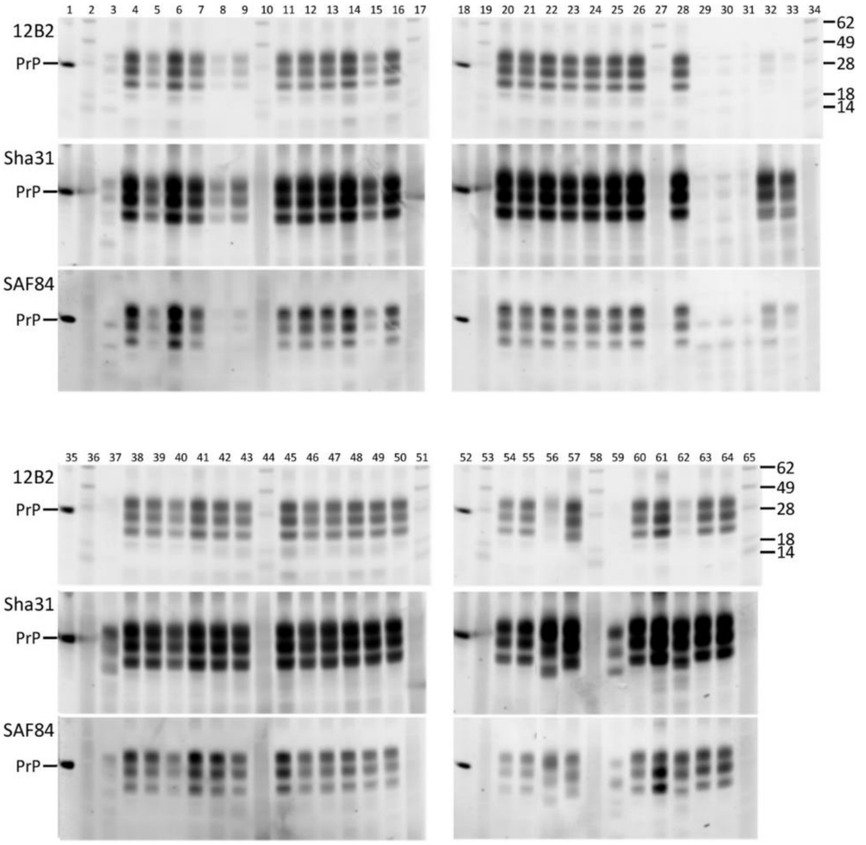

Lanes:

- 1, 18, 35, 52: rec-ovPrP$_{ARQ}$ (15 ng)
- 3, S2
- 4, S3
- 5-7, N1
- 8-9, N12
- 11-13, N3
- 14-16, F3
- 20-21, F10
- 22-24, F11
- 25, 26, 28, F14
- 29-31, F16
- 32, 33, 37, goat BSE
- 38-39, C1
- 40-41, C2
- 42, 43, 45, UK-B2
- 46-47, UKA2
- 48-49, UKD2
- 50, 54-55, G3
- 56, ovine BSE
- 57, ovine scrapie
- 59, goat CH1641
- 60, 87A
- 61, 79A
- 62, 301C
- 63, ME7
- 64, 22C

Representative triplex WB showing 21K/19K discrimination in RIII mice infected with goat TSE isolates or mouse-adapted scrapie strains, as indicated in the legend on the right of the blots. Samples from sheep with scrapie (lane 57), sheep with BSE (lane 56) and goat experimentally infected with CH1641 (lane 59) are included for comparison. In each blot, 15 ng of rec ovine PrP-ARQ were loaded, in order to determine the amount of PrP^{res} signals in ng per mg brain tissue. Replica blots were detected with mAbs 12B2, Sha31 or SAF84, as indicated on the left of the blots. Note that only RIII mice infected with goat BSE, as well as samples from sheep with BSE and goat with CH1641, show strongly reduced signal with 12B2.

Supplementary Figure S3. Triplex WB results in RIII mice.

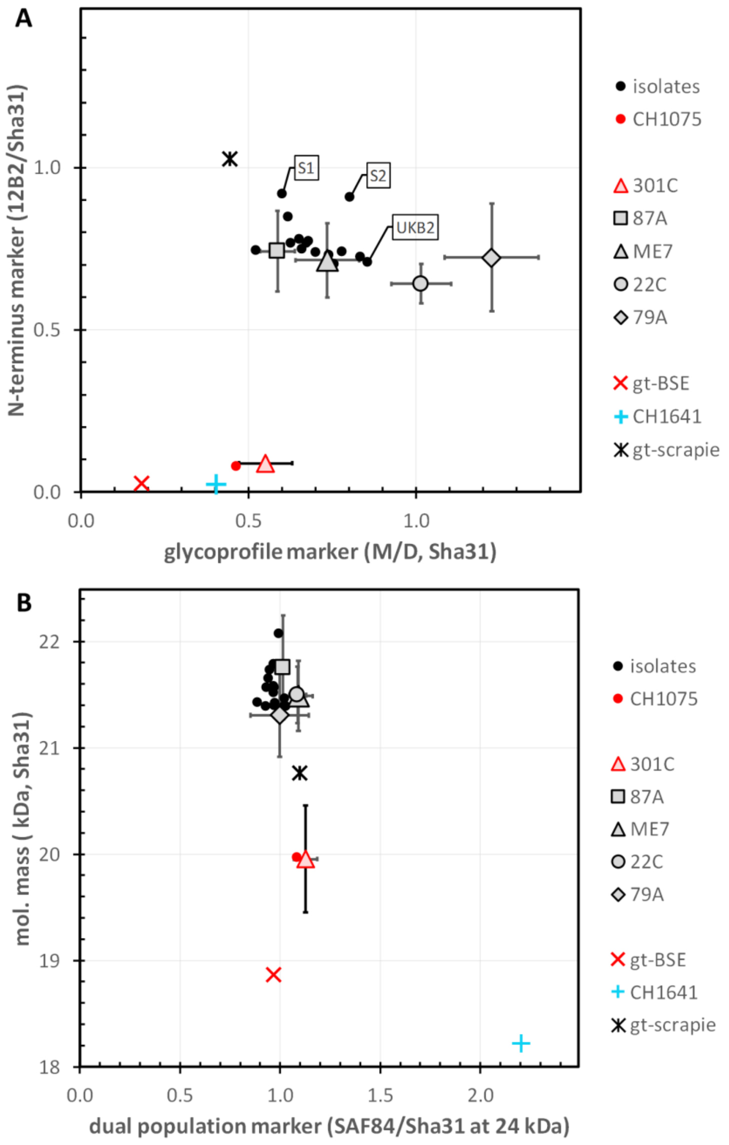

Dotplots showing the 87A/ME7 scrapie like behaviour of the 15 isolates from RIII mice with quantifiable PrP^{res} signals in the Western blot of figure S2, probed with antibodies 12B2, Sha31 and SAF84 in a mix. Panel A shows the glycoprofile marker (M/D ratio with mAb Sha31) on the X-axis, and relative N-terminal epitope content (12B2/Sha31 total signal ratio) on Y-axis; panel B shows the dual population marker (SAF84/Sha31 ratio at 24 kDa) and the molecular mass of non-glycosylated band respectively on X- and Y-axis. Circular filled symbols represent the RIII mice inoculated with the field study cases (black), including goat BSE sample CH1075 (red). Per isolate, the averaged result from the inoculated mice in figure S2 is shown. Reference samples (see dot-plot legend for symbol explanation): strains 87A, ME7, 22C, 79A and 301C (from RIII mice) have been presented together with their standard deviations. Furthermore, samples of experimental goat BSE (n=10), experimental small ruminant CH1641 (n=3, 2 sheep, 1 goat) and goat scrapie (n=18) show their position in relation to the RIII samples. All groups of RIII mice inoculated with field goat TSEs were not deviating from the 87A/ME7 references: neither a BSE like (low 12B2/Sha31; low kDa) nor a CH1641-like (low 12B2/Sha31; dual PrPres population value >1.25) TSE-type could be shown to be present in any of the RIII mice. Nevertheless, cases S1 and S2 were peculiar in showing a high N-terminus epitope content, and UKB2 the most high M/D glycoprofile marker value (see boxes in A).

Supplementary Figure S4. Western blot analysis of PrP^{Sc} in Bv109M.

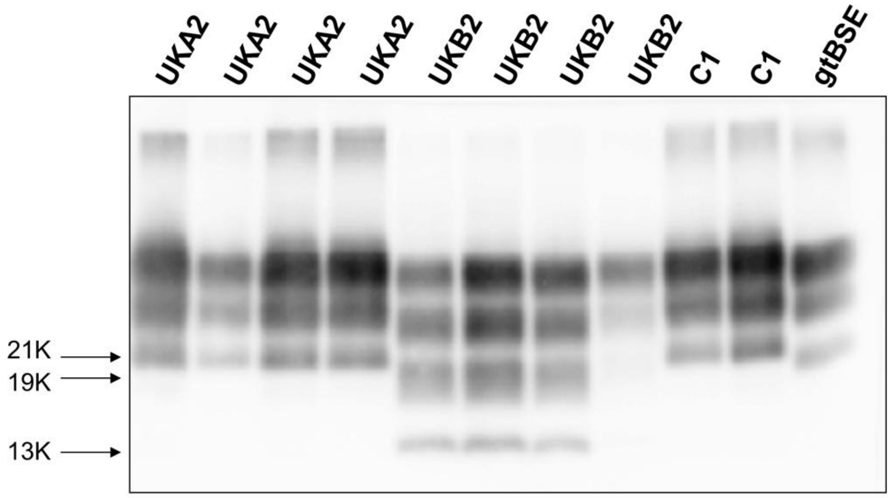

Representative WB of PrP^{res} in individual Bv109M infected with the TSE isolates UKA2, UKB2, C1 or goat BSE, as indicated on the top of the blot. PrP^{res} was detected with the C-terminal mAb with SAF84, which allows to recognise all the PrP^{res} fragments detected in Bv109M infected with goat TSEs (i.e. 21K, 19K and 13K, whose position of the unglycosylated fragment is indicated at the left of the blot). Note that Bv109M infected with UKA2 and C1 scrapie isolates propagated 21K PrP^{res}, while the Bv109M with goat BSE shows 19K PrP^{res}. Among the 4 Bv109M infected with UKB2, 3 show 19K accompanied by an additional 13K PrP^{res}, and one shows only 19K PrP^{res}.

Supplementary Figure S5. TE profiles with all rodent models (upper panel) or after exclusion of tg-shVRQ and tg-bov (lower panel).

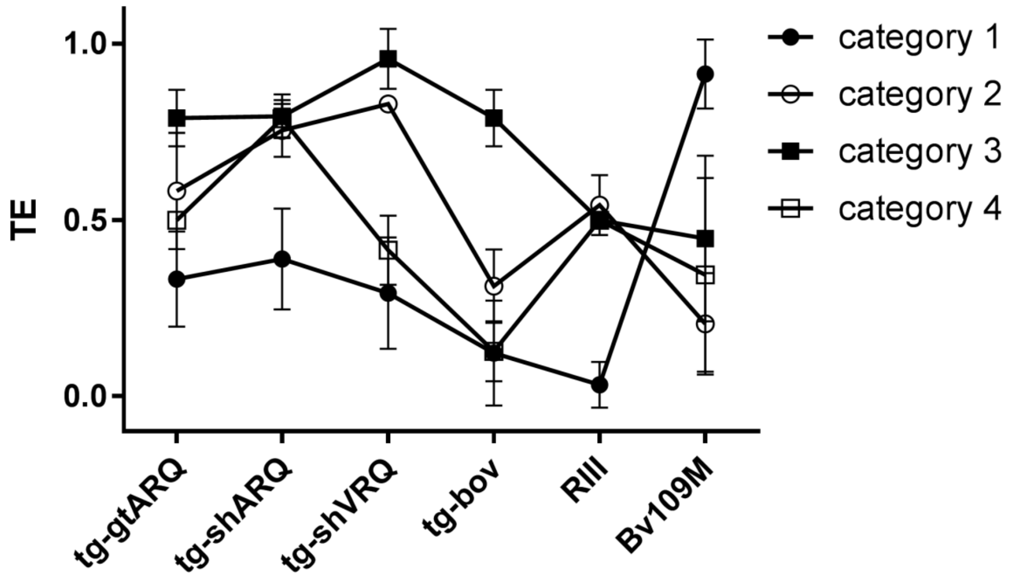

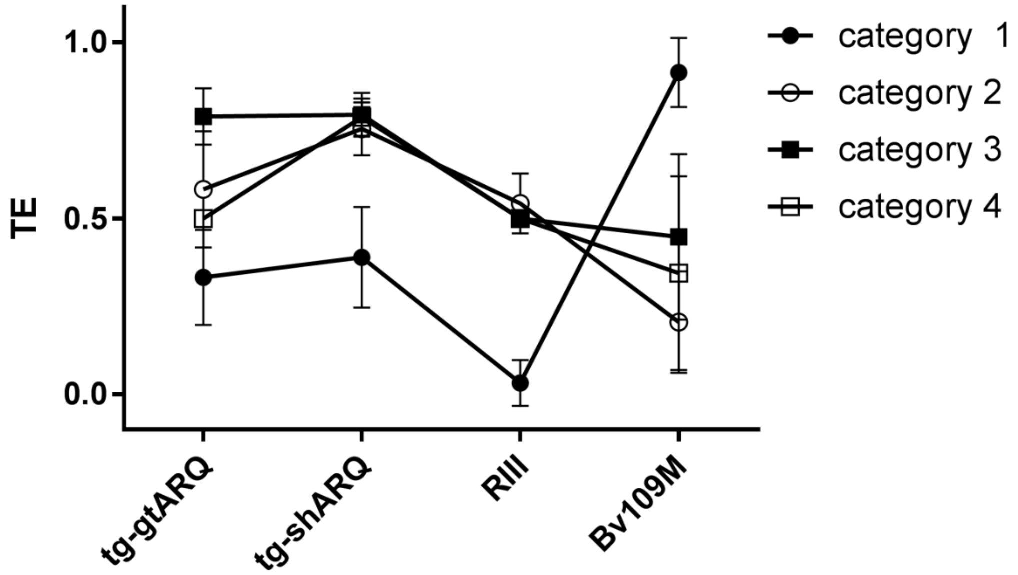

The comparison shows that after the exclusion of tg-shVRQ and tg-bov datasets, categories 2-to-4 have overlapping TE profiles, while category 1 remains a distinct group. Thus, tg-shVRQ and tg-bov data account for the differentiation among categories 2-to-4.

Supplementary Figure S6. Discriminatory IHC in different brain regions of case UKB2..

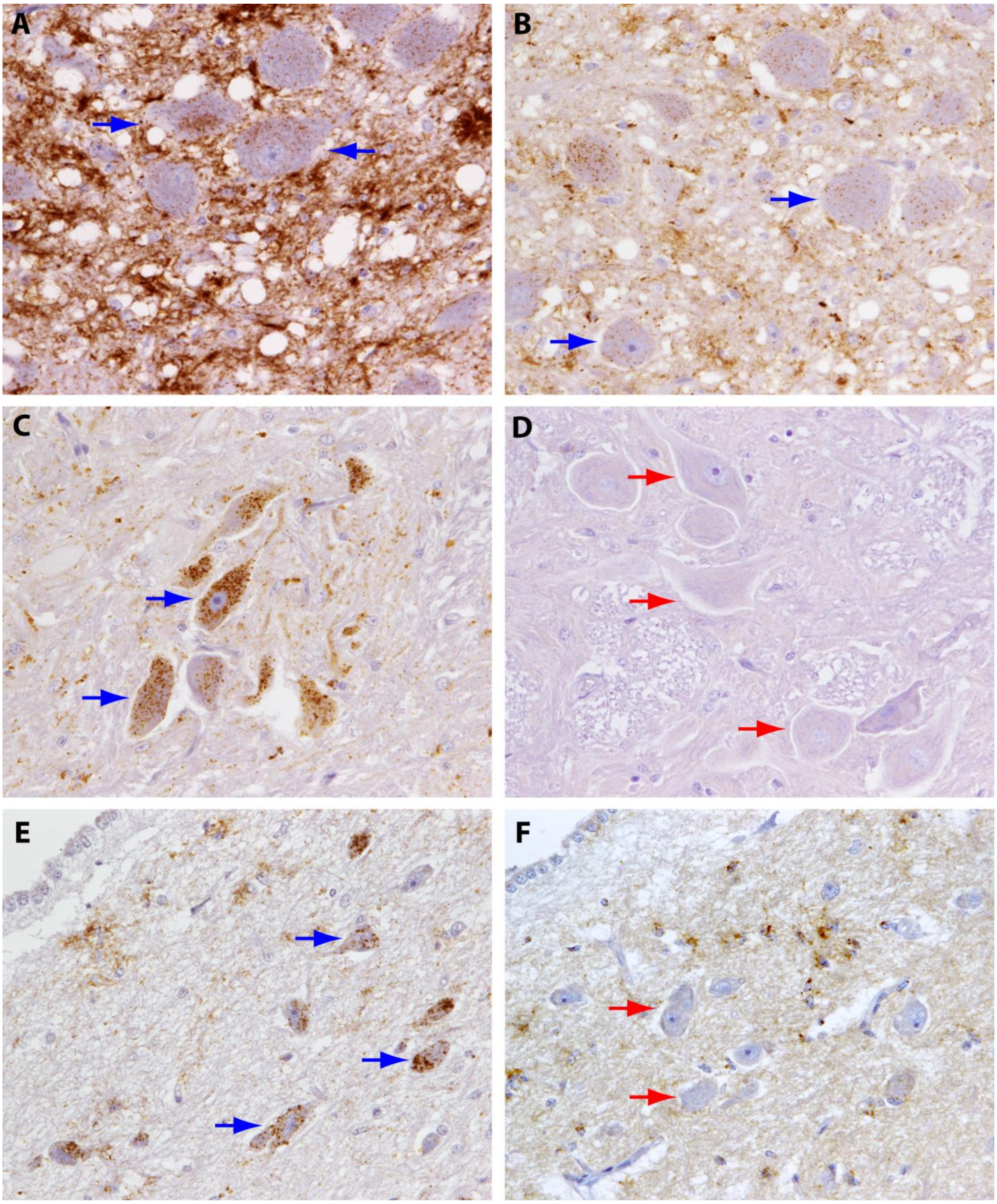

Coronal brain sections were labelled with monoclonal antibodies R145 or P4. The dorsal motor nucleus of the vagus was the only area where intraneuronal labelling was evident with both antibodies (R145 (A); P4 (B)). This labelling is compatible with classical scrapie. In other nuclei at the same coronal level (obex), such as the cuneate nuclei, PrP^{Sc} was identified only with R145 (C) whilst no labelling was evident in sections that were treated with P4 (D). This pattern is compatible with BSE or CH1641. In other brain areas PrP^{Sc} that was readily detectable with R145 (E, parietal cortex) was not evident when the sections were labelled with P4 (F, parietal cortex); labelling in the neuropil was variable. This combination is compatible with BSE or CH1641. Blue arrows indicate neurons with intraneuronal labelling; red arrows indicate neurons devoid of intraneuronal labelling.

Supplementary Figure S7. Uncropped original blots used for Fig.4A.

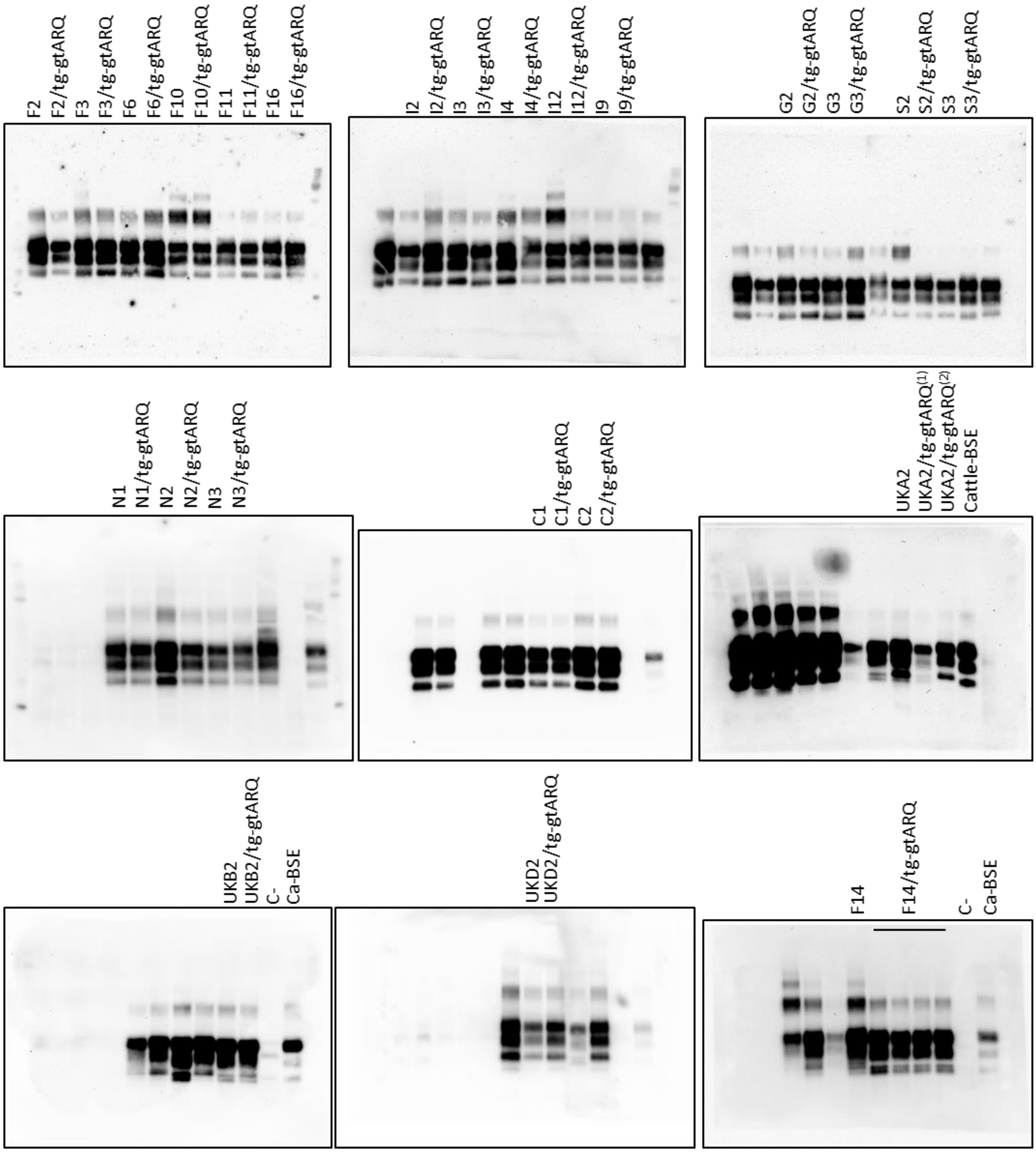

Original uncropped blots from which cropped lanes were used in Figure 4. The indicated lanes are those cropped and presented in Fig. 4A, where they are indicated with the same label used here.

Supplementary Figure S8. Uncropped original blots used for Fig.4A-C.

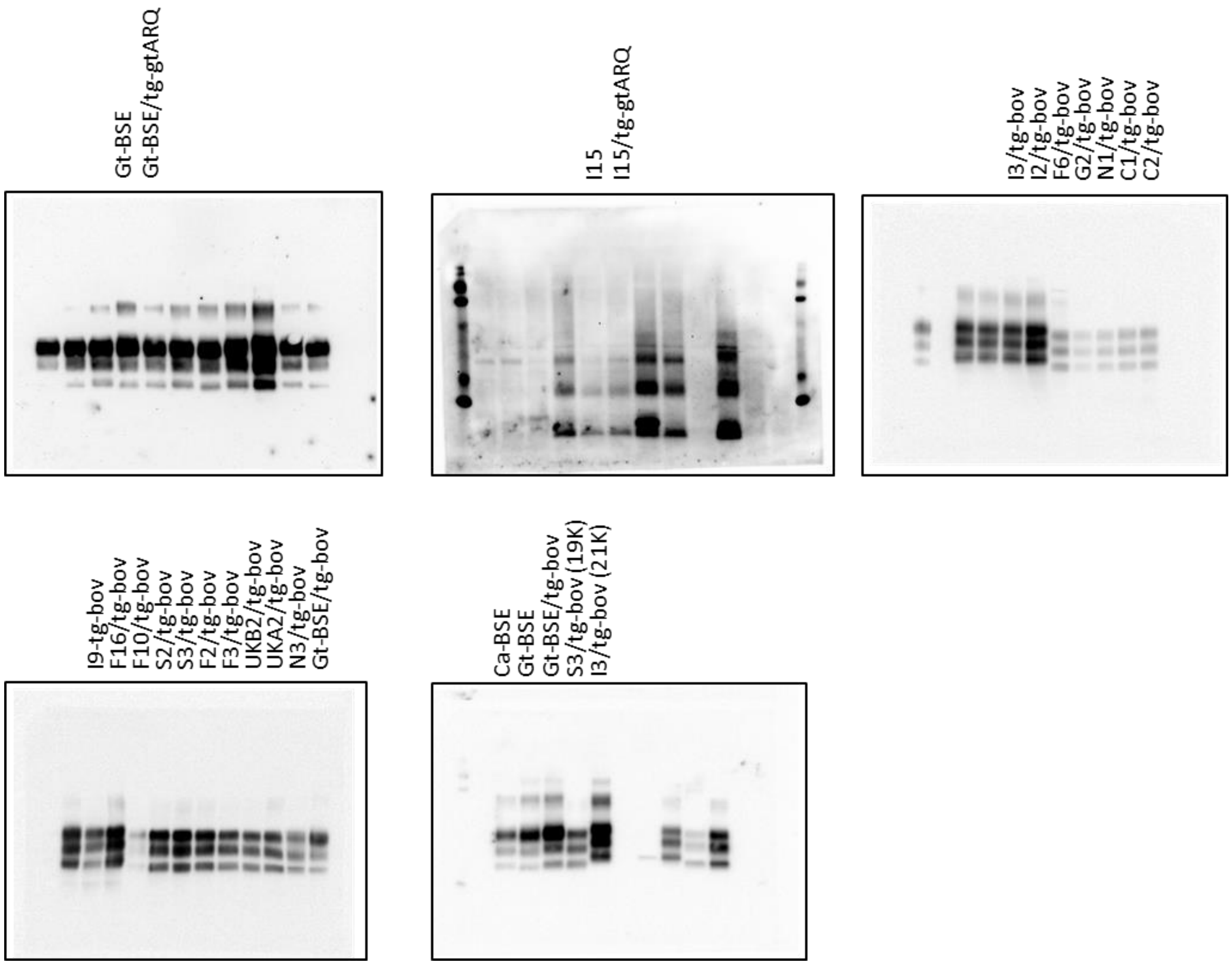

Original uncropped blots from which cropped lanes were used in Figure 4. The indicated lanes are those cropped and presented in Fig. 4A, 4B and 4C, where they are indicated with the same label used here.
